# Maternal nutrition and offspring lung health: sex-specific pathway modulation in fibrosis, metabolism, and immunity

**DOI:** 10.29219/fnr.v69.11035

**Published:** 2025-01-03

**Authors:** Shuangyi Zhao, Zhimin Chen, Huina Liu, Xinyan Wang, Xiuru Zhang, Huirong Shi

**Affiliations:** 1 Department of Obstetrics, The First Affiliated Hospital of Zhengzhou University, Zhengzhou, China; 2 Department of Surgery of Spine and Spinal Cord, Henan Provincial People’s Hospital, Zhengzhou, China; 3 Department of Gynaecology, The First Affiliated Hospital of Zhengzhou University, Zhengzhou, China

**Keywords:** lung injury, maternal diet, offspring, RNA-seq, high salt diet

## Abstract

**Background:**

Maternal nutrition profoundly influences offspring health, impacting both prenatal and early postnatal development. Previous studies have demonstrated that maternal dietary habits can affect key developmental pathways in the offsprings, including those related to lung function and disease susceptibility. However, the sex-specific impact of a maternal high-salt diet (HSD) on offspring lung injury remains poorly understood.

**Objective:**

This study aimed to investigate the sex-specific effects of maternal HSD on lung injury in mouse offsprings, focusing on pathways related to fibrosis, metabolism, immunity, and apoptosis.

**Design:**

Pregnant C57BL/6J mice were subjected to either normal or HSD conditions during gestation. Lung tissues from the male and female offsprings were analyzed using high-throughput RNA sequencing and bioinformatics tools to examine transcriptomic changes. Wet-lab validation, including Masson trichrome staining, immunofluorescence for α-SMA, and qRT-PCR for fibrotic markers (α-SMA, collagen I, Fn1, and TGF-β), was conducted to confirm fibrosis and other injury markers. Lung structure and weight were also evaluated to assess physical alterations due to maternal diet.

**Results:**

Maternal HSD significantly altered lung transcriptomes in a sex-specific manner. Male offsprings showed increased susceptibility to fibrosis, as confirmed by histological and molecular analyses, including elevated expression of α-SMA, collagen I, Fn1, and TGF-β. In contrast, female offsprings exhibited distinct changes in metabolic and immune pathways. These findings highlight the differential regulation of pulmonary injury mechanisms between male and female offsprings exposed to HSD.

**Conclusions:**

Maternal HSD induces sex-specific lung injury in offsprings by disrupting critical pathways involved in fibrosis, metabolism, immunity, and apoptosis. The combination of transcriptomic and orthogonal data underscores the need for balanced maternal nutrition during pregnancy to promote long-term respiratory health in offsprings. These results provide new insights into the sex-specific vulnerabilities to lung disease arising from maternal diet.

## Popular scientific summary

- How a High-Salt Diet During Pregnancy Can Affect Offspring’s Lung Health.
- What a mother eats during pregnancy can have lasting effects on her child’s health, even influencing how their lungs develop. In this study, we explored how a high-salt diet (HSD) during pregnancy impacts the lung health of offspring, with surprising differences between males and females.
- Using mice, we compared the effects of a balanced diet to a high-salt diet during pregnancy. After the offspring were born, we analyzed their lung tissues using RNA sequencing technology to study gene expression patterns related to lung disease, metabolism, immunity, and cell death.
- The results were striking: a high-salt diet caused significant changes in lung health, but these effects were different for male and female offspring. For example, male offspring were more prone to a type of lung infection called pulmonary candidiasis, while females were more likely to experience pneumothorax (collapsed lung). Genes related to metabolism, immunity, fibrosis (lung scarring), and cell death were also affected differently based on sex.
- This research highlights that a mother’s diet can shape her child’s lung health and disease risk in a sex-specific way. It emphasizes the importance of balanced nutrition during pregnancy to help ensure long-term lung health for children.

High-salt diets (HSD) are a significant feature of Western dietary patterns and have been recognized as a major contributor to global morbidity and mortality (1–3). Despite growing public awareness and efforts to modify dietary behaviors, the worldwide sodium consumption continues to far exceed the physiological requirements for health (4, 5). The World Health Organization (WHO) recommends that adults, including pregnant and lactating women, limit their daily salt intake to less than 5 g (6). However, in many countries, sodium intake consistently surpasses these guidelines, with processed foods contributing up to 75% of dietary sodium in Europe and North America, while in Japan and China, significant sodium intake comes from salt and soy sauce used in home-cooked meals (5, 7). This excessive intake of dietary sodium poses a growing global health challenge, as it has been implicated in the development of cardiovascular diseases (8, 9), hypertension (10–14), and neurodegenerative diseases (15, 16).

In addition to its systemic effects, HSD has been shown to affect organ-specific functions, including the lungs. Studies suggest that a maternal high-fat diet can impair pulmonary angiogenesis (17) and disrupt early lung signaling pathways (18). HSD, in particular, has been associated with heightened inflammation (19) and impaired airway responsiveness (20, 21). However, there is a critical gap in understanding the impact of maternal high-salt intake on lung development in offsprings, and whether this effect varies between male and female offsprings. Maternal nutrition, including HSD, significantly influences fetal development, and previous studies have demonstrated sex-specific differences in the prenatal response to environmental stressors (22–26). Males are generally more prone to neonatal respiratory distress syndrome (RDS) and early-life respiratory infections, while females tend to exhibit increased susceptibility to autoimmune and inflammatory lung diseases later in life (27–29). These biological differences suggest that the effects of prenatal dietary stressors, including maternal HSD, may manifest differently in male and female offsprings.

Given the systemic inflammatory and immune-modulatory effects of HSD (30–33), it is plausible that maternal HSD could induce sex-specific lung injury in offsprings, affecting key developmental pathways related to fibrosis, metabolism, immunity, and apoptosis. Despite growing awareness of the influence of the maternal diet on offspring health, this area remains underexplored, particularly with respect to lung development.

We hypothesize that maternal consumption of a HSD leads to sex-specific lung injury in mouse offsprings, altering critical pathways related to fibrosis, metabolism, immunity, and apoptosis. To test this hypothesis, we subjected pregnant C57BL/6J mice to HSD and analyzed lung tissues from male and female offsprings through high-throughput RNA sequencing (RNA-seq), immunofluorescence, and quantitative reverse transcription polymerase chain reaction (qRT-PCR). Our study aims to 1) investigate the morphological lung abnormalities and sex-specific differences in the offsprings, and 2) elucidate the impact of HSD on gene expression and signaling pathways involved in fibrosis, metabolism, immunity, and apoptosis.

## Materials and methods

### Animals and experimental groups

**Fig. 1:**
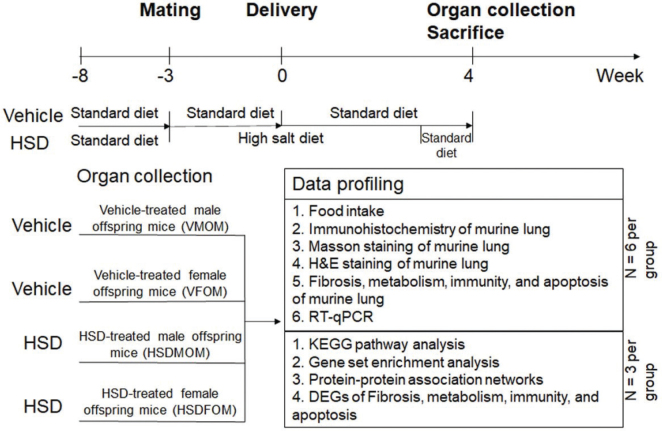
Experimental design and data profiling workflow. The timeline outlines the experimental process from mating to organ collection and data profiling. Pregnant C57BL/6J mice were divided into two dietary groups: one receiving a standard diet (Vehicle) and the other a high-salt diet (HSD) from the third week of pregnancy until delivery. Offspring lung tissues were collected 4 weeks post-birth and divided into four groups: vehicle-treated male offspring (VMOM), vehicle-treated female offspring (VFOM), HSD-treated male offspring (HSDMOM), and HSD-treated female offspring (HSDFOM).

Adult male and female C57BL/6J mice (10–12 weeks) were obtained from GemPharmatech Co. Ltd (Jiangsu, China). Mice were allowed to get acclimatized for 2 weeks before the initiation of the breeding program. The flowchart of the experiment was shown as Fig. 1. Mice were housed in a cage in a ratio of one male to two females and fed standard chow (American Institute of Nutrition [AIN]-93G containing 0.26% NaCl) or a 4% HSD (AIN-93G with additional increase in total to 4% NaCl) after mating as previously reported (25, 34, 35). Diet compositions are provided in Supplementary Table 1. Mating was confirmed by the presence of a vaginal plug, marking gestation day 0. Pregnant mice were subjected to a HSD for the first 3 weeks of gestation, after which they were switched to a vehicle diet (standard chow) for the remainder of their pregnancy. The day of birth was designated as postnatal day 0. After full-term delivery, one male and one female pup were randomly selected from each female’s offspring at 4 weeks of age for experimental analysis. Six offsprings were selected from one of the three different litters, including three males and three females for RNA-seq (*n* = 3); the same method was used to select the offsprings of mice used for immunohistochemistry (*n* = 6). The offsprings were sacrificed at 4 weeks old, and the experimental groups were divided as follows: (1) Vehicle-treated male offspring mice (VMOM), where pregnant dams were fed a standard chow diet; (2) HSD-treated male offspring mice (HSDMOM), where pregnant dams were fed a 4% HSD for 3 weeks; (3) Vehicle-treated female offspring mice (VFOM) , where pregnant dams were fed a standard chow diet; and (4) HSD-treated female offspring mice (HSDFOM), where pregnant dams were fed a 4% HSD for 3 weeks. In total, 12 offsprings were used for RNA-seq (*n* = 3 per sex per treatment) and 24 offsprings for immunohistochemistry (*n* = 6 per sex per treatment). This selection ensures that both sexes and the effects of maternal diet are adequately represented. The study was reviewed and approved by the Institutional Animal Care and Use Committee with approval No. XF20190628, and all methods were carried out in accordance with relevant guidelines and regulations. This study was carried out in compliance with the ARRIVE guidelines.

### Immunohistochemistry of murine lung

All immunohistochemistry and immunofluorescence of lung tissues from VMOM, HSDMOM, VFOM, and HSDFOM groups were performed according to a previously published protocol (25, 36–38). Briefly, mice were anaesthetized with pentobarbital sodium by intraperitoneal injection and cervical dislocation prior to lung removal. Sections of lung tissues from VMOM, HSDMOM, VFOM, and HSDFOM groups were deparaffinized and rehydrated in xylene I-III (10023418, Sinopharm Chemical Reagent Co., Ltd., Shanghai, China), 75–100% ethanol (100092683, Sinopharm Chemical Reagent Co., Ltd., Shanghai, China), and distilled water. After antigen repair, endogenous peroxidase blocking and serum blocking, anti-CD3 antibody (GB11014, Servicebio Company, Wuhan, China), anti-F4/80 antibody (GB113373, Servicebio Company, Wuhan, China), and anti-cleaved caspase-3 antibody (GB11009-1, Servicebio Company, Wuhan, China) prepared in PBS (pH 7.4, G0002, Servicebio Company, Wuhan, China) at 1:300 were added dropwise to the tissue sections, and the sections were incubated flat at 4°C overnight in a wet box. Then, after washing the sections with PBS, secondary antibodies (G23303/GB21303, Servicebio Company, Wuhan, China) were added to cover the tissue and incubated at room temperature for 50 min. Finally, after treating with DAB (G1211, Servicebio Company, Wuhan, China), hematoxylin (G1004, Servicebio Company, Wuhan, China), and dehydration in 75–100% ethanol and xylene I-III, the sections were examined by microscopy and image acquisition for analysis using a light microscope with a slide scanner (Pannoramic 250/MIDI, 3D HISTECH Ltd., Hungary) and ImageJ software was used (version 1.53k, Wayne Rasband, National Institutes of Health, United States).

### Masson staining of murine lung

Masson staining of murine lung was performed according to a previously published protocol (39, 40). Briefly, sections of lung tissues from VMOM, HSDMOM, VFOM, and HSDFOM groups were deparaffinized and rehydrated in xylene I-III (10023418, Sinopharm Chemical Reagent Co., Ltd., Shanghai, China), 75–100% ethanol (100092683, Sinopharm Chemical Reagent Co., Ltd., Shanghai, China), and distilled water. Sections were treated with Masson A-F from Masson’s trichrome staining kit (G1006, Servicebio Company, Wuhan, China), rinsed with 1% glacial acetic acid, and dehydrated with anhydrous ethanol. Finally, the sections were treated with anhydrous ethanol and xylene for 5 min, and then sealed with neutral gum (10004160, Sinopharm Chemical Reagent Co., Ltd., Shanghai, China). The sections were examined by microscopy and image acquisition for analysis was done using an orthotopic light microscope (Nikon ECLIPSE E100, Nikon, Japan) and ImageJ software (version 1.53k, Wayne Rasband, National Institutes of Health, United States).

### H&E staining of murine lung

Hematoxylin–eosin (H&E) staining of the murine lung was performed according to a previously published protocol (25, 40–42). Lung tissues from VMOM, HSDMOM, VFOM, and HSDFOM groups were carefully prepared to minimize potential artifacts. The tissues were initially fixed in 10% neutral buffered formalin and then embedded in paraffin. The paraffin-embedded sections were cut into 5 µm thick slices using a microtome. The sections were deparaffinized in three changes of xylene (Xylene I-III, Sinopharm Chemical Reagent Co., Ltd., Shanghai, China) to remove the paraffin and then rehydrated through a graded ethanol series (75–100%, Sinopharm Chemical Reagent Co., Ltd., Shanghai, China) followed by distilled water. Next, the sections were stained in hematoxylin solution for 3–5 min to visualize the nuclei, followed by a brief rinse in distilled water. They were then stained in eosin solution for 5 min to counterstain the cytoplasm. After staining, the sections were dehydrated through a graded ethanol series (75–100%) and cleared in three changes of xylene (Xylene I-III). Finally, the sections were mounted with neutral gum (Sinopharm Chemical Reagent Co., Ltd., Shanghai, China) and covered with coverslips. The stained sections were examined under a Nikon ECLIPSE E100 light microscope (Nikon, Japan), and images were captured for analysis. Image analysis was performed using ImageJ software (version 1.53k, Wayne Rasband, National Institutes of Health, United States). Lung injury scores were used to quantify changes in lung structure in the VMOM, VFOM, HSDMOM, and HSDFOM groups as previously described (43). According to the bronchiole and peri-bronchial infiltration, bronchiole and bronchial exudation, perivascular infiltration, edema, atelectasis and other conditions, the degree of microscopic injury score was calculated.

### RNA extraction and RNA sequencing

Lung tissues from VMOM, HSDMOM, VFOM, and HSDFOM groups were collected, weighed, and stored at −80°C. RNA-seq was performed in the Beijing Genomics Institute (BGI) and total RNA of four groups was extracted using Trizol reagent (Invitrogen Inc, Carlsbad, CA, USA) as previously reported (25, 44). Briefly, total RNA of lung tissues was obtained by pellet air drying for 5–10 min in a biological safety cabinet (Airstream, Class II, A2 type; Esco, Philadelphia, USA) after grinding, washing, and centrifugation. Total RNA was lysed using DEPC-treated water and then qualified and quantified using a Nano Drop and Agilent 2100 bioanalyzer (Thermo Fisher Scientific, MA, USA). Obtained RNA was purified by oligo(dT)-attached magnetic beads and fragmented into pieces smaller than 200 bp using Mg_2_^+^. The first strand of cDNA was synthesized using random hexamer primers and reverse transcription. The second strand was synthesized using buffer, dNTPs, RNase H, and DNA polymerase I. Subsequently, the cDNA underwent end-repair, 3’ adenylation, and ligation with sequencing adapters. PCR amplification was performed on the cDNA fragments obtained from the previous step, and the products were purified by Ampure XP Beads and then dissolved in EB solution. For quality control, it was validated on an Agilent Technologies 2100 bioanalyzer (Agilent Technologies, Palo Alto, Calif.). After denaturing the PCR products to a single-stranded state, the cyclization reaction system is prepared and mixed thoroughly for a certain time at appropriate temperature to obtain single-stranded cyclic products. Single-stranded cyclic DNA molecules were replicated into DNA nanoballs (DNBs) and loaded onto a BGISEQ-500 platform (BGI Group, Shenzhen, China) for sequencing. Single-end sequencing was conducted with a read length of 50 bp, ensuring a sequencing depth of 20 million reads per sample. Raw sequencing data underwent quality control using SOAPnuke software (version 1.5.2, https://github.com/BGI-flexlab/SOAPnuke) to remove low-quality reads and adapters. Clean reads were ensured to have Q20 and Q30 scores above 90%. Clean reads were mapped to the reference genome using Bowtie2 (version 2.2.5, https://sourceforge.net/projects/bowtiebio) and HISAT2 (version 2.0.4, http://www.ccb.jhu.edu/software/hisat/index.shtml). This ensured accurate alignment and coverage. Gene expression levels were quantified using RSEM (RNA-Seq by Expectation-Maximization). Counts were normalized to FPKM (Fragments Per Kilobase of transcript per Million mapped reads) to account for library size and sequencing depth.

### Real-time RT-PCR

Total RNA was extracted from frozen lung tissues using Trizol reagent (Invitrogen Inc, Carlsbad, CA, USA) (25, 44, 45). Then, a total of 2.5 μg of RNA were subjected to reverse transcription using the MightyScript First Strand cDNA Synthesis Master Mix (BBI Life Sciences Corporation, China) to prepare cDNA according to the manufacturer’s instructions. Quantitative RT-PCR was performed with SGExcel FastSYBR Mixture (Sangon Biotech, China). Each sample was examined in triplicate, and β-actin was used as an internal control. The primers for the real-time PCR were synthesized by Sangon Biotech, China. The primer sequences were as follows: α-SMA, forward 5’-GGCTCTGGGCTCTGTAAGG-3’, reverse 5’-CTCTTGCTCTGGGCTTCATC-3’; Collagen α1 (I), forward 5’-GCCCGAACCCCAAGGAAAAGAAGC-3’, reverse 5’-CTGGGAGGCCTCGGTGGACATTAG-3’; Fn1, forward 5’-GTGTAGCACAACTTCCAATTACGAA-3’, reverse 5’-GGAATTTCCGCCTCGAGTCT-3’; TGF-β1, forward 5’-TTGCTTCAGCTCCACAGAGA-3, reverse 5’-GTTGGACAACTGCTCCACCT-3’; β-actin, forward 5’-AGGCCAACCGTGAAAAGATG-3’, reverse 5’-AG AGCATAGCCCTCGTAGATGG-3’.

### Functional annotation with Kyoto Encyclopedia of Genes and Genomes and gene ontology

Pathway terms and function terms of differential expression genes (DEGs) (|FC| > 1.5 and *FDR* < 0.05) were discovered based on the Kyoto Encyclopedia of Genes and Genomes (KEGG) database and Gene Ontology (GO) database for VMOM, HSDMOM, VFOM, and HSDFOM groups. We used the software package phyper (https://stat.ethz.ch/R-manual/R-devel/library/stats/html/Hypergeometric.html) based on R to calculate *P* values, and then performed a multiple test to obtain correct *P* values using software package *q*-values (https://bioconductor.org/packages/release/bioc/html/qvalue.html). *Q* value (corrected *P* value) <= 0.05 was the threshold value, and the term that met this condition was defined as the KEGG pathway and GO term that were significantly enriched in the candidate genes.

### Protein-protein association networks

The interaction between genes and genes of mouse offspring’s lung involved in metabolic genes and immune genes, protein–protein association networks (PPANs) functional enrichment analysis was determined using the STRING database (version 11.0, https://string-db.org/) (46). The interaction sources include textmining, experiments, databases, co-expression, neighborhood, gene fusion, and co-occurrence, minimum required interaction score was set as high confidence (0.700), max number of interactors to show was set as no more than five interactors, network type was set as full STRING network (the edges indicate both functional and physical protein associations), and kmeans clustering was adopted.

### Transcription factors

To predict the transcription factors (TFs) regulating genes associated with fibrosis, metabolism, immunity, and apoptosis, we utilized the ChIP-X Enrichment Analysis 3 (ChEA3) tool (47). The TFs were ranked using the integration of MeanRank and Fisher’s exact test *P* value, with the top five TFs displayed.

### Statistical analysis

All data are shown as mean ± standard error of the mean (SEM). The comparison between male and female mice was analyzed using the two-way analysis of variance (ANOVA), followed by Bonferroni post-hoc tests. Statistical significance was set at *P* < 0.05. The bar charts, graphs, and data analysis were performed using SPSS 27.0 software (IBM) and GraphPad Prism 8 software (La Jolla, USA). Heatmaps were created in R package (64-bit, version 3.5.3).

## Results

### HSD causes lung injury of mouse offsprings

**Fig. 2:**
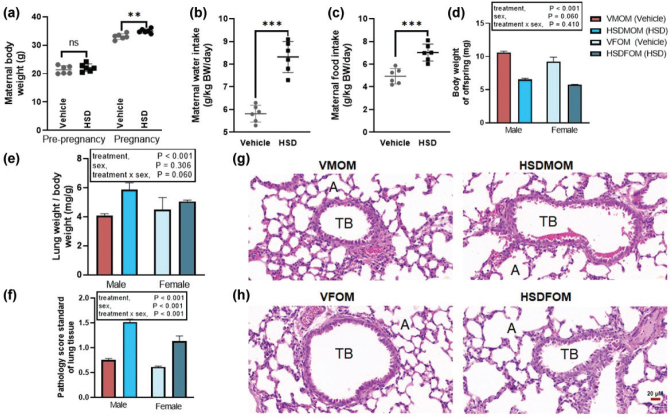
The effect of HSD on sex-specific lung weight and structure of mouse offsprings. (a) Body weight before pregnancy and during pregnancy of vehicle and HSD treated group. ***P < 0.001. (b) Maternal water intake of vehicle and HSD treated group. ***P < 0.001. (c) Maternal food intake of vehicle and HSD treated group. ***P < 0.001. (d) Weight of body obtained from vehicle-treated male offspring mice (VMOM), HSD-treated male offspring mice (HSDMOM), Vehicle-treated female offspring mice (VFOM), and HSD-treated female offspring mice (HSDFOM). (e) Weight of lung obtained from VMOM, HSDMOM, VFOM, and HSDFOM. (f) Score of lung tissue pathology for VMOM, HSDMOM, VFOM, and HSDFOM. (g–h) Histopathology of the lung tissue obtained from VMOM, HSDMOM, VFOM, and HSDFOM. *N* = 6 individual mice per group. The scale bar represents 20 µm. HSD, High-salt diets. TB, Terminal bronchiole. A, Alveoli. VMOM, Vehicle-treated male offspring mice.

To investigate the effect of HSD on lung weight and the structure of male mouse offsprings and female mouse offsprings, the weight, HE, and the corresponding damage score was noted for the lung tissues of mice offsprings treated with a normal diet and HSD according to the previous method (43, 48). Firstly, we assessed the effect of HSD on maternal weight, water intake and food consumption. As show in Fig. 2a–c, the effects of HSD during pregnancy on maternal body weight gain, food and water intakes were also analyzed. There were statistically significant differences in weight gain, food intake, and water intake between the two groups. Secondly, in prenatal salt-exposed offsprings, the mouse offsprings had decreased body weight compared to the vehicle-treated group. A two-way ANOVA demonstrated a significant main effect of HSD (*P* < 0.001), but not sex (*P* = 0.060) and sex × HSD interaction (*P* = 0.410) on body weight (Fig. 2d). Again, in prenatal salt-exposed offsprings, the mouse offsprings had increased ratios of lung mass and body weight compared to the vehicle-treated group. A two-way ANOVA demonstrated a significant main effect of HSD (*P* < 0.001), but not sex (*P* = 0. 306) and sex × HSD interaction (*P* = 0.060) on ratios of lung mass and body weight (Fig. 2e). Finally, we evaluated the lung injury caused by HSD. Alveolar wall thickening and erythrocyte sludge were particularly evident in the offsprings of the male mice. Animals in the vehicle group scored significantly lower than those in the HSD group. A significant sex × HSD interaction (*P* < 0.001) as well as a significant main effect of HSD (*P* < 0.001) and sex (*P* < 0.001) on pulmonary fibrosis (Fig. 2f–h) were noted. These results suggest that maternal HSD caused injury to the lungs of the mouse offsprings.

### HSD alters sex-specific transcriptome composition of mouse offspring’s lung

**Fig. 3:**
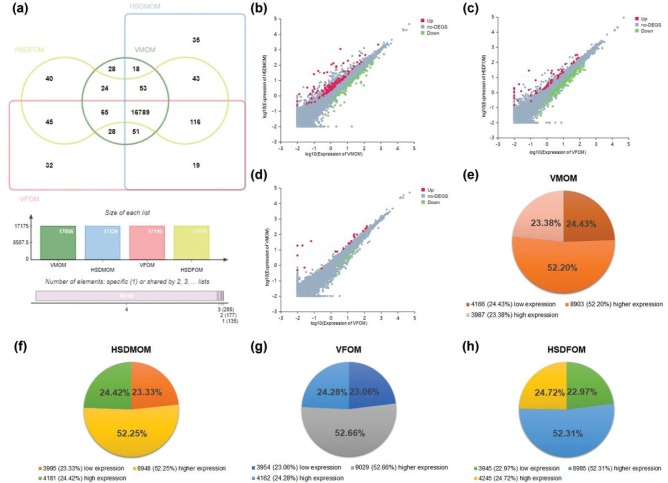
The effect of HSD on sex-specific transcriptome composition of mouse offspring’s lung. (a) Venn plot illustrating the distribution of genes identified in the lungs of vehicle-treated male offspring mice (VMOM), HSD-treated male offspring mice (HSDMOM), vehicle-treated female offspring mice (VFOM), and HSD-treated female offspring mice (HSDFOM). Unique genes for VMOM and HSDMOM were 145 and 213, respectively, with 16,911 shared genes. For female offspring, unique genes were 130 for VFOM and 160 for HSDFOM, with 17,015 shared genes. (b–d) Scatter plot showing differentially expressed genes (DEGs) between groups: (b) VMOM versus HSDMOM, (c) VFOM versus HSDFOM, and (d) VMOM versus VFOM. Up-regulated DEGs are indicated by red dots (|FC| > 2 and *FDR* < 0.05), down-regulated DEGs by green dots (|FC| > 2 and *FDR* < 0.05), and genes without significant differences by gray dots. (e–h) displaying the composition of lung transcriptome by expression levels: (e) VMOM, (f) HSDMOM, (g) VFOM, and (h) HSDFOM. Gene expression categories are defined as low expression genes (0 < FPKM < 1), higher expression genes (1 ≤ FPKM < 20), and high expression genes (FPKM ≥ 20). *N* = 3 individual mice per group.

To investigate the impact of HSD on lung transcriptome composition in male and female mouse offsprings, high-throughput RNA-Seq was performed (25). The Venn diagram (Fig. 3a) reveals the distribution of genes across the groups, indicating that in male offsprings, 145 genes were unique to VMOM, and 213 were unique to HSDMOM, with a total of 16,911 shared genes. In female offsprings, 130 genes were unique to VFOM, and 160 to HSDFOM, with 17,015 shared genes. We also compared the number of genes unique in the offsprings of male and female mice in the vehicle-treated group to 123 and 212, respectively, and the number of genes for the shared genes was 16,933 (Fig. 3a).

Scatter plots further illustrate the differentially expressed genes (DEGs) between the groups. In male offsprings, 112 DEGs were up-regulated, and 81 were down-regulated when comparing VMOM to HSDMOM (Fig. 3b and Supplementary Table 2). In female offsprings, 59 DEGs were up-regulated, and 164 were down-regulated in the comparison of VFOM to HSDFOM (Fig. 3c and Supplementary Table 3). Furthermore, between male and female offsprings in the normal diet-treated group, we identified 21 up-regulated and 17 down-regulated DEGs (Fig. 3d and Supplementary Table 4).

Finally, to further analyze the effect of HSD on the lung transcriptome of mouse offsprings, we further analyzed its composition based on its expression level. As per the reported rules, the transcripts were divided into three categories: low expression genes (0 < FPKM < 1), higher expression genes (1 ≤ FPKM < 20), and high expression genes (FPKM ≥ 20) (25, 49). In male mouse offsprings, the composition ratios of transcripts showed differences between the vehicle-treated and HSD-treated groups: low expression genes (VMOM vs. HSDMOM, 4,166 [24.43%] vs. 3,995 [23.33%]), higher expression genes (8,903 [52.20%] vs. 8,948 [52.25%]), and high expression genes (3,987 [23.38%] vs. 4,181 [24.42%]) (Fig. 3e, f and Supplementary Tables 5–6). Similarly, in female offsprings, the composition ratios were: low expression genes (VFOM vs. HSDFOM, 3,954 [23.06%] vs. 3,945 [22.97%]), higher expression genes (9,029 [52.66%] vs. 8,985 [52.31%]), and high expression genes (4,162 [24.28%] vs. 4,245 [24.72%]) (Fig. 3g, h and Supplementary Tables 7–8). Collectively, these findings indicate that HSD significantly alters the transcriptome composition in the lungs of mouse offsprings, with distinct effects observed between male and female offsprings.

### HSD alters the KEGG pathway of mouse offspring’s lung

**Fig. 4:**
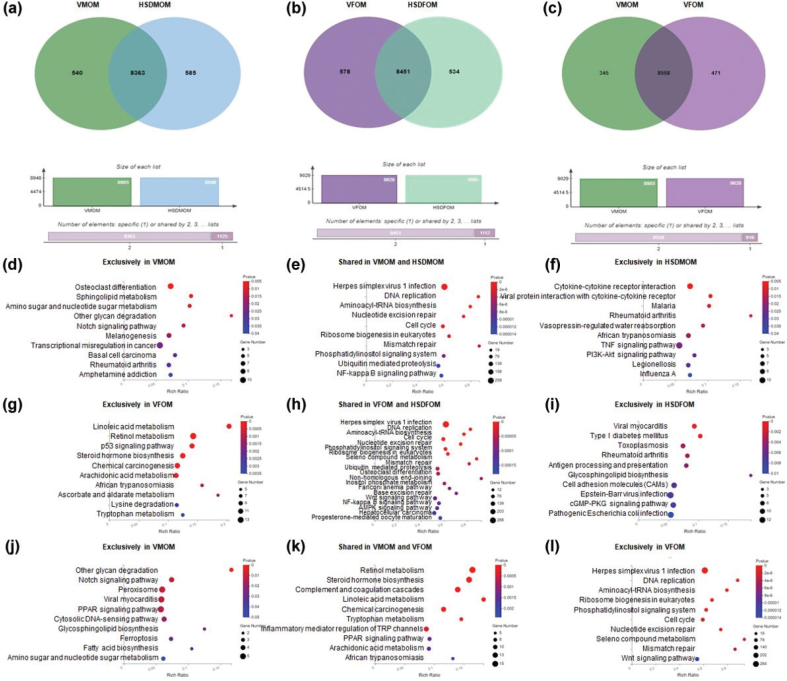
The effect of HSD on the KEGG pathway of male and female offspring’s lung. (a) Venn plot of higher expression genes obtained from VMOM and HSDMOM. (b) Venn plot of higher expression genes obtained from VFOM and HSDFOM. (c) Venn plot of higher expression genes obtained from VMOM and VFOM. (d) Top 10 KEGG pathways enriched in higher expression genes unique to VMOM with *P* < 0.05. (e) Top 10 KEGG pathways enriched in higher expression genes shared in VMOM and HSDMOM with *P* < 0.05. (f) Top 10 KEGG pathways enriched in higher expression genes unique to HSDMOM with *P* < 0.05. (g) Top 10 KEGG pathways enriched in higher expression genes unique to VFOM with *P* < 0.05. (h) Top 20 KEGG pathways enriched in higher expression genes shared in VFOM and HSDFOM with *P* < 0.05. (i) Top 10 KEGG pathways enriched in higher expression genes unique to HSDFOM with *P* < 0.05. (j) Top 10 KEGG pathways enriched in higher expression genes unique to VMOM with *P* < 0.05. (k) Top 10 KEGG pathways enriched in higher expression genes shared in VMOM and VFOM with *P* < 0.05. (l) Top 10 KEGG pathways enriched in higher expression genes unique to VFOM with *P* < 0.05. *N* = 3 individual mice per group. HSD, High-salt diets. VMOM, Vehicle-treated male offspring mice. HSDMOM, HSD-treated male offspring mice. VFOM, Vehicle-treated female offspring mice. HSDFOM, HSD-treated female offspring mice. KEGG, Kyoto Encyclopedia of Genes and Genomes.

To investigate the effects of HSD on the composition of higher expressed transcripts and associated KEGG pathways in the lung of mouse offsprings, we used the Venn plot and KEGG enrichment analyses. In male mouse offsprings, higher expression genes unique to vehicle-treated and HSD-treated offsprings are 540 and 585, respectively; higher expression genes shared in vehicle-treated and HSD-treated offsprings are 8,363 (Fig. 4a). In female mouse offsprings, higher expression genes unique to vehicle-treated and HSD-treated offsprings are 578 and 534, respectively; higher expression genes shared in vehicle-treated and HSD-treated offsprings are 8,451 (Fig. 4b). We also compared that higher expression genes unique to male and female offsprings with normal diet are 345 and 471, respectively; higher expression genes shared in male and female offsprings with normal diet are 8,363 (Fig. 4c). In the male offsprings, 13, 28, and 13 significant KEGG pathways (*P* < 0.05) were discovered in the unique to VMOM group (Fig. 4d and Supplementary Table 9), shared VMOM and HSDMOM groups (Fig. 4e and Supplementary Table 10), HSDMOM group (Fig. 4f and Supplementary Table 11), respectively. In the female offsprings, 12, 35, and 10 significant KEGG pathways (*P* < 0.05) were discovered in the unique to VFOM group (Fig. 4g and Supplementary Table 12), shared VFOM and HSDFOM groups (Fig. 4h and Supplementary Table 13), HSDFOM group (Fig. 4i and Supplementary Table 14), respectively. Finally, we also further compared the differences in the KEGG pathways enriched in unique and shared higher expression genes between male and female mouse offsprings. A total of 10, 13, and 15 significant KEGG pathways (*P* < 0.05) were discovered in the unique to VMOM group (Fig. 4j and Supplementary Table 15), shared VMOM and VFOM groups (Fig. 4k and Supplementary Table 16), and unique to VFOM group (Fig. 4l and Supplementary Table 17), respectively.

### HSD induces KEGG pathways in male and female mouse offsprings with different pulmonary diseases

**Fig. 5:**
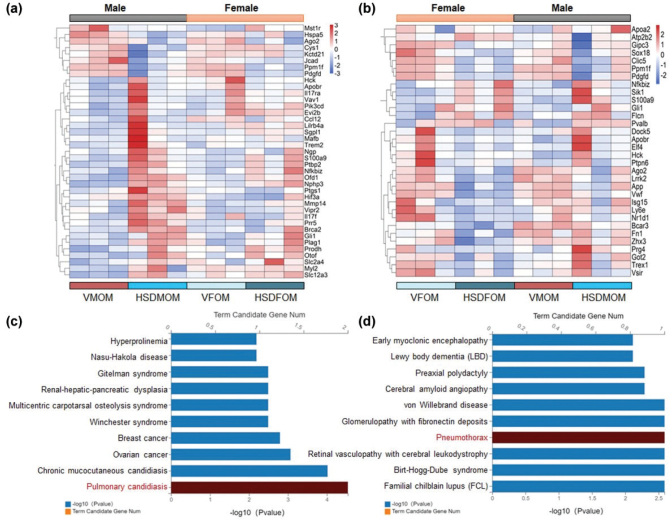
HSD causes different pulmonary diseases in male and female mouse offsprings. (a) Heatmap of pulmonary diseases associated DEGs (|FC| > 1.5 and *FDR* < 0.05) in male mouse offsprings. (b) Heatmap of pulmonary diseases associated DEGs (|FC| > 1.5 and *FDR* < 0.05) in female mouse offsprings. (c) KEGG disease of pulmonary diseases associated DEGs in male mouse offsprings. (d) KEGG disease of pulmonary diseases associated DEGs in female mouse offsprings. *N* = 3 individual mice per group. KEGG, Kyoto Encyclopedia of Genes and Genomes. DEGs, differentially expressed genes.

To investigate whether HSD affects lung health, we first examined the expression levels of differential genes associated with pulmonary disease. In the male mouse offsprings, 39 differential genes were generated for HSD as compared to the vehicle-treated offsprings (Fig. 5a); however, in female mouse offsprings, HSD produced 32 differential genes (Fig. 5b). Furthermore, we performed KEGG disease enrichment analysis on these differential genes, and the results showed that HSD could significantly induce male mouse offsprings to contract pulmonary candidiasis (*P* = 2.90E-05) (Fig. 5c), while HSD significantly induced female mouse offsprings to contract pneumothorax (*P* = 2.58E-03) (Fig. 5d). These results showed that HSD could significantly change the lung health of mouse offsprings and induce mouse offsprings to form sex-specific pulmonary diseases.

### HSD alters the expression profile of fibrosis in mouse offspring’s lung

**Fig. 6:**
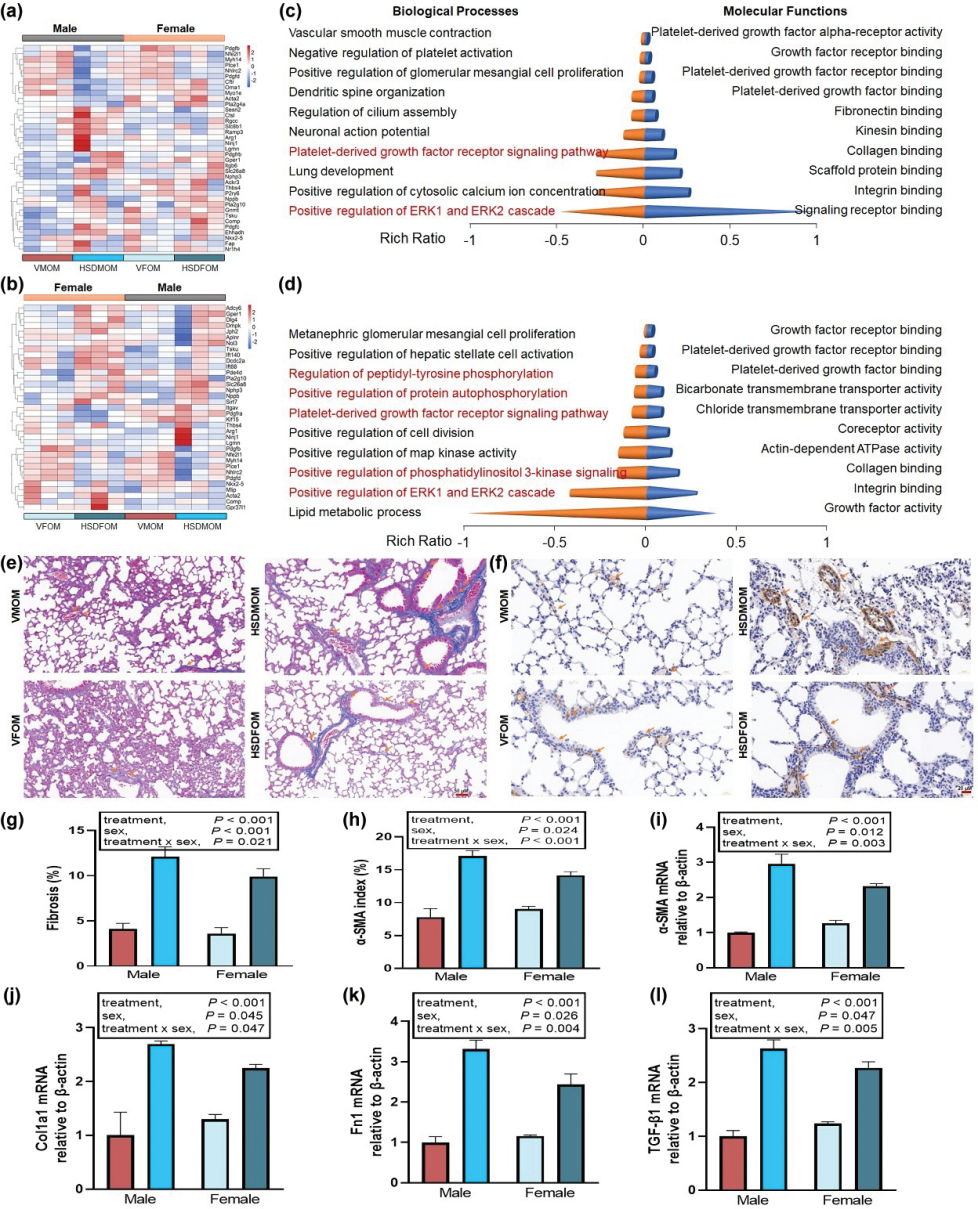
The effect of HSD on pulmonary fibrosis in male and female mouse offsprings. (a) Heatmap of pulmonary fibrosis associated DEGs (|FC| > 1.5 and *FDR* < 0.05) in male mouse offsprings. (b) Heatmap of pulmonary fibrosis associated DEGs (|FC| > 1.5 and *FDR* < 0.05) in female mouse offsprings. (c) Top 10 biological processes and molecular functions of pulmonary fibrosis associated DEGs in male mouse offsprings. (d) Top 10 biological processes and molecular functions of pulmonary fibrosis associated DEGs in female mouse offsprings. *N* = 3 individual mice per group. (e) Masson staining of the lung tissue obtained from VMOM, HSDMOM, VFOM, and HSDFOM. The scale bar represents 50 μm. *N* = 6 individual mice per group. (f) Measurement of the expression level of α-SMA by immunohistochemical staining (*n* = 6 per group) obtained from VMOM, HSDMOM, VFOM, and HSDFOM treated lung tissue. The scale bar represents 20 μm. (g) Quantitative assess the fibrosis content of Masson staining obtained from VMOM, HSDMOM, VFOM, and HSDFOM treated lung tissue. (h) Quantitative assess the α-SMA content of immunohistochemical staining obtained from VMOM, HSDMOM, VFOM, and HSDFOM treated lung tissue. (i–l) qRT-PCR analysis of α-SMA, cola1a, Fn1, and TGF-β mRNA expression in lung tissues (*n* = 6 per group).

To study whether HSD will affect lung health, we investigated the expression of differential genes related to pulmonary fibrosis. Compared with the offsprings of mice treated with normal diet, HSD produced 39 differential genes related to pulmonary fibrosis in the offsprings of male mice (Fig. 6a). However, in the offsprings of female mice, HSD produced 32 differential genes related to pulmonary fibrosis (Fig. 6b). Furthermore, we conducted GO enrichment analysis on these differential genes, and the results showed that HSD formed different biological processes and molecular functions in male and female mouse offsprings (Fig. 6c, d and Supplementary Table 18). In male mouse offsprings, a total of 12 significantly molecular functions (*P* < 0.001) were discovered and were divided into five categories: 1) binding: integrin binding (*P* = 1.10E-04), collagen binding (*P* = 2.80E-04), platelet-derived growth factor binding (*P* = 1.99E-04), platelet-derived growth factor receptor binding (*P* = 1.12E-08), and growth factor receptor binding (*P* = 2.84E-07); 2) catalytic activity: actin-dependent ATPase activity (*P* = 9.66E-04), phospholipase A2 activity (*P* = 9.66E-04), and phospholipase A2 activity consuming 1,2-dioleoylphosphatidylethanolamine (*P* = 9.66E-04); 3) molecular function regulator: growth factor activity (*P* = 1.51E-04); 4) molecular transducer activity: coreceptor activity (*P* = 9.66E-04); 5) transporter activity: chloride transmembrane transporter activity (*P* = 4.59E-04) and bicarbonate transmembrane transporter activity (*P* = 3.61E-04) (Fig. 6c and Supplementary Table 18). In female mouse offsprings, a total of 32 significantly molecular functions (*P* < 0.01) were discovered and were divided into four categories: 1) binding: platelet-derived growth factor receptor binding (*P* = 1.92E-06), fibronectin binding (*P* = 1.86E-05), growth factor receptor binding (*P* = 7.56E-05), kinesin binding (*P* = 7.95E-05), platelet-derived growth factor binding (*P* = 1.78E-04), collagen binding (*P* = 2.36E-04), scaffold protein binding (*P* = 2.88E-04), signaling receptor binding (*P* = 5.67E-04), integrin binding (*P* = 1.67E-03), P2Y1 nucleotide receptor binding (*P* = 1.67E-03), growth factor activity (*P* = 2.11E-03), protein binding (*P* = 2.12E-03), opsonin binding (*P* = 3.34E-03), death effector domain binding (*P* = 5.01E-03), protein kinase C binding (*P* = 5.78E-03), beta-1 adrenergic receptor binding (*P* = 6.68E-03), neuroligin family protein binding (*P* = 6.68E-03), and C-X3-C chemokine binding (*P* = 8.34E-03); 2) catalytic activity: platelet-derived growth factor alpha-receptor activity (*P* = 1.67E-03), NAD-dependent histone deacetylase activity (H3-K18 specific) (*P* = 1.67E-03), beta-fructofuranosidase activity (*P* = 3.34E-03), oligo-1,6-glucosidase activity (*P* = 3.34E-03), protein-succinyllysine desuccinylase activity (*P* = 3.34E-03), protein-glutaryllysine deglutarylase activity (*P* = 3.34E-03), phosphoric diester hydrolase activity (*P* = 4.24E-03), arginase activity (*P* = 5.01E-03), calcium- and calmodulin-responsive adenylate cyclase activity (*P* = 8.34E-03), and hydrolase activity acting on carbon–nitrogen (but not peptide) bonds, in linear amidines (*P* = 8.34E-03); 3) molecular function regulator: myosin phosphatase regulator activity (*P* = 6.68E-03); 4) molecular transducer activity: apelin receptor activity (*P* = 1.67E-03), prosaposin receptor activity (*P* = 3.34E-03), and estrogen receptor activity (*P* = 8.34E-03) (Fig. 6d and Supplementary Table 18).

As previously reported, adverse environmental exposures such as hyperoxia (50) and methyl mercaptan (43) as well as pulmonary diseases such as chronic obstructive pulmonary disease (51) can cause pulmonary fibrosis. Systemic maternal inflammation (52) and maternally derived inflammatory mediators (53) are also important influences on pulmonary fibrosis in the offsprings, and preventing cytokine expression has the potential to reduce the deleterious consequences of perinatal inflammation on lung development and subsequent function (including apoptosis) in the offsprings (54, 55). Therefore, we further used immunohistochemical staining and quantitative reverse transcription polymerase chain reaction (qRT-PCR) to assess whether maternal HSD causes pulmonary fibrosis in the mouse offsprings. As shown in Fig. 6e and g, compared with the vehicle-treated group, maternal HSD causes pulmonary fibrosis in the male and female mouse offsprings. A significant sex × HSD interaction (*P* = 0.021) as well as a significant main effect of HSD (*P* < 0.001) and sex (*P* < 0.001) on pulmonary fibrosis (Fig. 6g). The results of α-smooth muscle actin (α-SMA) staining shown that increased expression of α-SMA was identified in the HSD-treated male and female mouse offsprings compared with the vehicle-treated group (Fig. 6f and h). Additionally, four key fibrotic markers including α-SMA, type I collagen, fibronectin and TGF-β1 mRNA expression was then confirmed using qRT-PCR analysis in lung tissue samples. As shown in Fig. 6i–l, the increased α-SMA, type I collagen (Col1a1), fibronectin (Fn1), and TGF-β1 mRNA expression were identified in the HSD-treated male and female mouse offsprings compared with the vehicle-treated group.

Finally, to predict which TFs regulate fibrosis-associated transcripts, the ChEA3 tool was used. The top five TFs in the lungs of male mouse offsprings after HSD treatment were MTF1, SPI1, IRF8, TFEC, and IRF5; and the top five TFs in the lungs of female mouse offsprings were and TBX5, NKX26, ZBED6, AEBP1, and MEOX1. These results showed that HSD could form different degrees of pulmonary fibrosis in the offsprings of mice of different sexes.

### HSD alters expression profile of metabolism in mouse offspring’s lung

To investigate whether HSD affects lung metabolism, we examined the expression levels of differential genes associated with lung metabolism. In male offsprings, HSD produced 92 differential genes associated with lung metabolism, compared to the vehicle-treated offsprings (Supplementary Fig. 1A). However, in female offsprings, HSD produced 101 differential genes associated with lung metabolism, compared to the vehicle-treated offsprings (Supplementary Fig. 1B). Furthermore, KEGG enrichment analysis of these differential genes revealed that HSD formed different metabolic pathways in male and female offsprings. In male mouse offsprings, a total of 16 significant KEGG pathways (*P* < 0.001) were discovered (Supplementary Fig. 1C and Supplementary Table 19). In female mouse offsprings, a total of 19 significant KEGG pathways (*P* < 0.001) were discovered (Supplementary Fig. 1D and Supplementary Table 19). The Venn plot shows that the number of metabolism-associated DEGs unique to HSD-treated male offsprings and female offsprings are 77 and 86, respectively (Supplementary Fig. 1E). Then, protein–protein association networks (PPANs) reveal that the metabolism-associated DEGs have complex interactions in male and female offsprings. Three clusters of metabolism-associated DEGs were discovered in male mouse offsprings (Supplementary Fig. 1F), but two clusters of metabolism-associated DEGs were discovered in female mouse offsprings (Supplementary Fig. 1G). Gene set enrichment analysis (GSEA) further corroborated the activation of pathways linked to the positive regulation of carbon metabolism, glycine, serine and threonine metabolism-male, oxidative phosphorylation, and valine, leucine and isoleucine degradation in the metabolism following HSD treatment (Supplementary Fig. 1H–K). Finally, to predict which TFs regulate metabolism-associated transcripts, the ChEA3 tool was used. The top five TFs in the lungs of male mouse offsprings after HSD treatment were NR1I2, MLXIPL, ARID3C, CREB3L3, and NR1H4; and the top five TFs in the lungs of female mouse offsprings were and ARID3C, MLXIPL, NR1I3, NR1I2, and CREB3L3. Collectively, these results suggest that HSD has different effects on lung metabolism in offsprings of male and female mice.

### HSD alters expression profile of immunity in mouse offspring’s lung

To investigate whether HSD affects the lung immune environment, we examined the expression levels of lung immune-related differential genes. In male offsprings, HSD produced 77 lung immune-related differential genes compared to the vehicle-treated offsprings (Supplementary Fig. 2A); however, in female mouse offsprings, HSD produced 109 lung immune-related differential genes (Supplementary Fig. 2D). Furthermore, KEGG enrichment analysis of these differential genes revealed that HSD formed different immune pathways in male and female offsprings. In male mouse offsprings, a total of nine significant KEGG pathways (*P* < 0.05) were discovered and two KEGG pathways were unique, which were Th17 cell differentiation and T cell receptor signaling pathway (Supplementary Fig. 2B). In female mouse offsprings, a total of 12 significant KEGG pathways (*P* < 0.05) were discovered and the five KEGG pathways were unique, which were natural killer cell mediated cytotoxicity, complement and coagulation cascades, cytosolic DNA-sensing pathway, RIG-I-like receptor signaling pathway, and Fc gamma R-mediated phagocytosis (Supplementary Fig. 2C). The Venn plot shows that the number of immunity-associated DEGs unique to HSD-treated male offsprings and female offsprings are 56 and 88, respectively (Supplementary Fig. 2E). PPINs reveal that the immunity-associated DEGs have complex interactions in male and female offsprings. Two clusters of immunity-associated DEGs were discovered and different molecular functions were identified in male mouse offsprings (Supplementary Fig. 2F), but three clusters of immunity-associated DEGs were discovered and there were different molecular functions in female mouse offsprings (Supplementary Fig. 2G). The ChEA3 tool was used to predict which TFs regulate immune-associated transcripts. The top five TFs in the lungs of male mouse offsprings after HSD treatment were SPI1, MTF1, CEBPE, ZNF467, and SP110; and the top five TFs in the lungs of female mouse offsprings were and BATF2, PLSCR1, TFEC, IRF7, and BATF.

**Fig. 7:**
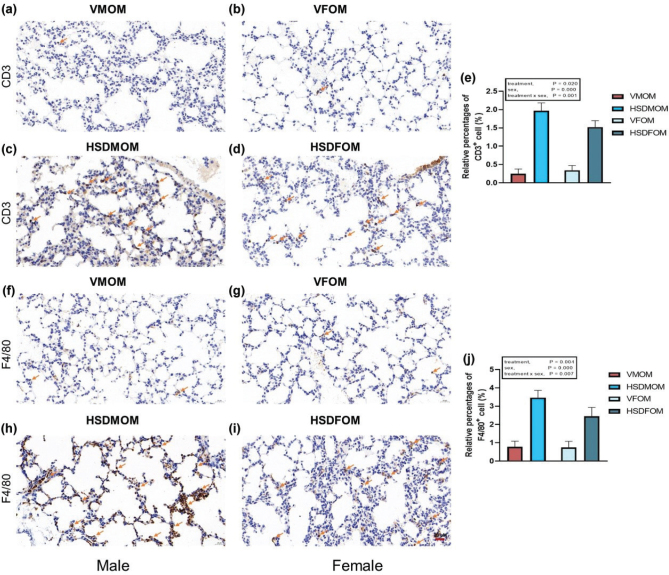
The effect of HSD on immunohistochemical staining in male and female mouse offsprings. (a) CD3 positive T cells of vehicle-treated male mouse offsprings. (b) CD3 positive T cells of normal treated female mouse offsprings. (c) CD3 positive T cells of HSD-treated male mouse offsprings. (d) CD3 positive T cells of HSD-treated female mouse offsprings. (e) Average relative abundance of CD3 positive T cells from vehicle- and HSD-treated lung tissue in male mice and female mouse offsprings. (f) F4/80 positive macrophages of vehicle-treated male mouse offsprings. (g) F4/80 positive macrophages of vehicle-treated female mouse offsprings. (h) F4/80 positive macrophages of HSD-treated male mouse offsprings. (i) F4/80 positive macrophages of HSD-treated female mouse offsprings. (j) Average relative abundance of F4/80 positive macrophages from vehicle- and HSD-treated lung tissue in male mice and female mouse offsprings. The scale bar represents 20 µm. *N* = 6 individual mice per group. HSD, High-salt diets.

Therefore, we further used immunohistochemical staining to assess whether maternal HSD causes inflammatory infiltration in the mouse offsprings. As shown in Fig. 7, compared with the vehicle-treated group, maternal HSD causes accumulation of CD3 positive T cells and F4/80 positive macrophages in the male and female mouse offsprings. A significant sex × HSD interaction (*P* < 0.001) as well as a significant main effect of HSD (*P* < 0.001) and sex (*P* = 0.030) was seen on CD3 positive T cells (Fig. 7a and c–f). A significant sex × HSD interaction (*P* = 0.004) as well as a significant main effect of HSD (*P* < 0.001) and sex (*P* = 0.001) was seen on F4/80 positive macrophages (Fig. 7b and g–j). Collectively, these results suggest that HSD has different effects on lung immunity of male and female mouse offsprings.

### HSD alters expression profile of apoptosis in mouse offspring’s lung

**Fig. 8:**
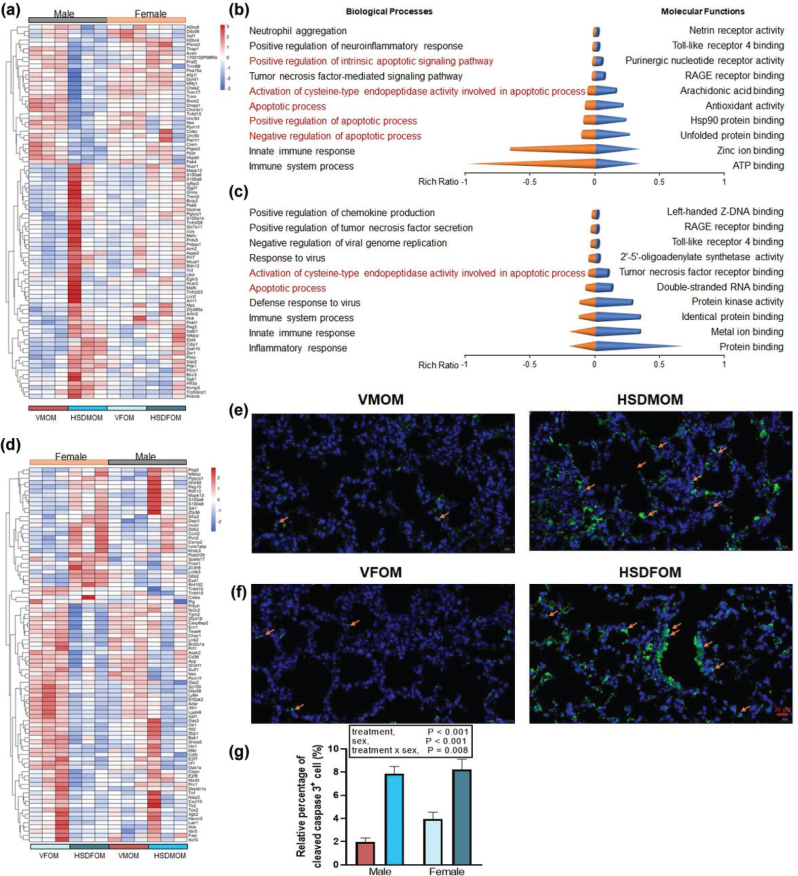
The effect of HSD on apoptosis in male and female mouse offsprings. (a) Heatmap of apoptosis associated DEGs (|FC| > 1.5 and *FDR* < 0.05) in male mouse offsprings. (b) Top 10 biological processes and molecular functions of apoptosis associated DEGs in male mouse offsprings with *Q* < 0.05. (c) Top 10 biological processes and molecular functions of apoptosis associated DEGs in female mouse offsprings with *Q* < 0.01. (d) Heatmap of apoptosis associated DEGs (|FC| > 1.5 and *FDR* < 0.05) in female mouse offsprings. *N* = 3 individual mice per group. (e) cleaved caspase-3^+^ cells of vehicle- and HSD-treated male mouse offsprings. (f) cleaved caspase-3^+^ cells of vehicle- and HSD-treated female mouse offsprings. (g) Average relative abundance of cleaved caspase-3^+^ cells from vehicle- and HSD-treated lung tissue in male mice and female mouse offsprings. The scale bar represents 20 µm. *N* = 6 individual mice per group. HSD, High-salt diets. DEGs, differentially expressed genes. The orange arrows in the figure represent typical positive cells.

To investigate whether HSD caused apoptosis, we examined the expression levels of differential genes associated with apoptosis in the lung. In male offsprings, HSD produced 86 differential genes associated with lung apoptosis, compared to the vehicle-treated offsprings (Fig. 8a). However, in female offsprings, HSD produced 88 differential genes associated with lung apoptosis when compared to the offsprings of mice treated with a normal diet (Fig. 8d). Furthermore, we conducted GO enrichment analysis on these differential genes, and the results showed that HSD formed different biological processes and molecular functions in male and female mouse offsprings (Fig. 8b, c and Supplementary Table 20). In male mouse offsprings, a total of 18 significantly molecular functions (*Q* < 0.05) were discovered (Fig. 8b and Supplementary Table 20). In female mouse offsprings, a total of 16 significantly molecular functions (*Q* < 0.01) were discovered (Fig. 8c and Supplementary Table 20). Then, to predict which TFs regulate apoptosis-associated transcripts, the ChEA3 tool was used. The top five TFs in the lungs of male mouse offsprings after HSD treatment were MXD1, MTF1, LTF, BATF2, and IRF9; and the top five TFs in the lungs of female mouse offsprings were and BATF2, PLSCR1, TFEC, BATF3, and IRF9. Finally, we further used immunofluorescent staining to assess whether maternal HSD causes apoptosis in the mouse offsprings. As shown in Fig. 8, compared with the vehicle-treated group, maternal HSD causes apoptosis in the male and female mouse offsprings. A significant sex × HSD interaction (*P* = 0.008) as well as a significant main effect of HSD (*P* < 0.001) and sex (*P* < 0.001) was seen on cleaved caspase-3^+^ cells (Fig. 8e–g). Collectively, these results indicate that HSD has different effects on the apoptosis of lung cells in the offsprings of male and female mice.

## Discussion

**Fig. 9:**
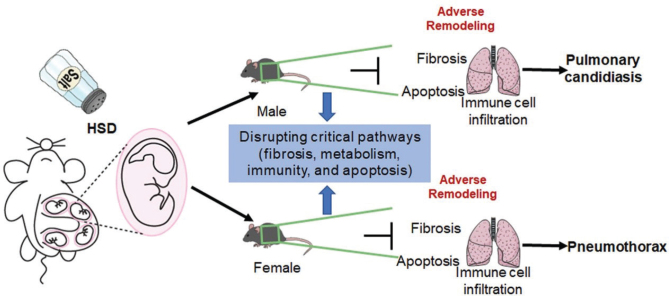
The adverse remodeling of HSD in lung. Maternal HSD induces sex-specific lung injury in offsprings by disrupting critical pathways involved in fibrosis, metabolism, immunity, and apoptosis.

As hypothesized, the significant finding of this study is that HSD can adversely affect the lung of mouse offsprings by altering fibrosis, metabolism, immunity, and apoptosis, and can induce sex-specific pulmonary injuries in mouse offsprings. The conclusions of this study are mainly based on the following findings: 1) HSD has different effects on the body weight of male and female offsprings in mice; 2) Consistent with previous reports, the transcriptional composition of the organs is sex-specific (25, 56–58). In this study, we found that the lungs of the male and female offsprings in mice had different transcriptome compositions, but the maternal HSD changed the transcriptome composition and produced different differential genes, KEGG pathway and GO terms for the male and female offsprings in mice; 3) HSD alters fibrosis, metabolism, immunity, and apoptosis in the lungs of the male and female offsprings in mice (Fig. 9). These findings will help us understand the negative effects of maternal HSD on the lungs of the male and female offsprings in mice.

There is evidence that maternal diets, such as those high in fat, salt, sugar and alcohol, may have an effect on the health of offsprings (59–62). The offsprings of mothers who consumed a high-fat diet showed an increase in airway resistance, even in those offsprings who consumed a normal diet after weaning (59). Maternal hypertension during pregnancy and a high-fat diet induced an enhanced hypertensive response to angiotensin II (ANG II) (60, 61). Fetal weight loss in offsprings of mothers on a high-fat diet was associated with catch-up growth, increased fat mass, and altered metabolic profile at weaning. Maternal metabolic parameters, fetal growth and development, metabolic status, and the fat axis at weaning are affected by a high fat and/or HSD (62). Prenatal exposure to an HSD affects the local renin-angiotensin system (RAS) and cardiac cells in the fetal heart in utero (63) and cardiac and vascular dysfunction in offsprings (64). We also found that maternal HSD resulted in weight loss in mouse offsprings and that male mouse offsprings lost more weight than female offspring mice. Moreover, maternal HSD sex-specific altered the lung cell size of the offspring mice.

Environmental exposures may have sexually dimorphic effects on lung development. Prenatal smog-exposed mice exhibit intrauterine and postnatal growth retardation and multiple alterations in the lung transcriptome and miRNA levels at E18.5 (65). Wild-type male offsprings had more pulmonary edema, lung injury, and neutrophil infiltration after hyperoxia exposure (66, 67). In the present study, we found that the lung development of the offspring mice was affected by maternal HSD, and the male offspring mice had more lung injury than the female offspring mice.

To date, there is a limited amount of data available on prenatal HSD and transcriptome changes in the lung. The current data mainly report on the effects of smoke exposure and exposure to hyperoxia on the transcriptome of the offspring of mice and their sexual dimorphism (65, 67). Consistent with previous reports (65, 68), the present study found that the lung transcriptome exhibited sexual dimorphism in both male and female offsprings and that maternal HSD exerted sex-specific effects. The number and type of highly expressed genes differed between male and female mouse offsprings, with the number of genes specific to female mouse offspring being ~1.4-fold higher than those specific to male mouse offsprings. Volcano plots showed that the number of lung transcriptomes and DEGs in male offsprings differed from those in female offsprings, respectively.

Fibrosis is a recognized as the cause of morbidity and mortality (69). Previous studies have shown that HSD significantly accelerates the formation of cardiac fibrosis in male and female mice and significantly alters the expression profile of fibrosis (70, 71). In children with Bronchopulmonary dysplasia (BPD), pathologic manifestations include fewer secondary septa and alveoli, fewer sites of emphysema, more fibrotic tissue, and widening and thickening of the interstitial spaces; fibrosis in ‘NEW’ BPD may also be an important contributor to abnormal lung function (72, 73). Pulmonary fibrosis is a component of many interstitial pulmonary diseases, such as idiopathic pulmonary fibrosis (74). Although high salt does not exacerbate bleomycin-induced pulmonary fibrosis in adult mice (75), we found that maternal HSD can cause pulmonary fibrosis in male and female mouse offsprings, and that male offsprings have higher pulmonary fibrosis than female offsprings.

High salt-induced diseases are caused by the interaction of genetic and dietary factors, such as kidney disease (76) and cardiovascular disease (25). High salt induces metabolic disorders, including changes in succinate, citrate and taurine in the urine of Wistar rats (77). Hypertension induced by high salt is considered a metabolic syndrome and increased dietary salt intake may be associated with metabolic disorders (78, 79). As previously reported consistently, HSD has been shown to disrupt cardiac (25, 80), plasma (81), muscle (82), hepatic (83), and renal (84) metabolic activities. We also found that maternal HSD does induce changes in the metabolic transcriptome composition of mouse offspring lungs; however, the exact mechanisms need to be further explored.

High salt intake is considered a potential modulator of inflammation and autoimmune diseases (21, 85, 86). HSD also plays a key role in the progression of autoimmune diseases, such as autoimmune encephalomyelitis, in which an HSD exerts its pathogenic function by modulating various immune responses (31, 87). HSD increases vascular pressure and triggers an immune response in the vasculature, leading to atherosclerosis and myocardial infarction (31). In addition, HSD has also been shown to modulate immune responses promoting breast tumor progression and lung metastasis (88). In this study, we demonstrated that HSD increased the infiltration of macrophages and T cells in the lungs of male and female mouse offsprings. Additionally, our analysis revealed that pathways involved in the immune response, such as IL-17 signaling pathway and antigen processing and presentation, show differential pathways between male and female offsprings, indicating sex-specific immune modulation in response to HSD. HSD dramatically altered the immune-related KEGG pathway in the lungs of male and female mouse offsprings.

Although our data have shown adverse effects of maternal HSD on lung structure and gene expression in the offspring mice, there are several limitations of this study that must be stated. First, this study was conducted only in C57BL/6J mice given 4% NaCl treatment in pregnant mice. The extent of damage to the offspring lungs needs to be further explored using higher salt concentrations. Second, the present study used a minimal number of samples for transcriptomics studies; therefore, further increases in the number of samples are needed for future studies, as well as for further investigation of the effects of maternal HSD on metabolites and proteomics in offspring mice. Third, this study was not performed to test lung function in mouse offsprings; therefore, future studies need to pay further attention to the effects of HSD on the physiological function of mouse lungs. Then, although this study primarily investigated the short-term effects of HSD on the offspring lung from a transcriptomic perspective, we recognize that observing its functional effects, long-term effects on lung development, and adult lung-associated diseases requires protein-level studies and further mechanistic research. To address this gap, future studies will integrate proteomics techniques to establish long-term observations to better understand how HSD alters lung structure and function in offsprings. Finally, the mouse model used in this study is nocturnal, which exhibits key differences from human behavior. Therefore, the clinical guidance implications of the findings of this study need to be viewed dialectically.

## Conclusions

Therefore, we conclude that maternal HSD has a sex-specific effect on the development of lungs in mouse offsprings. Maternal HSD induces significant alterations in the lung transcriptome, with male offsprings being more sensitive. Our data suggest that maternal HSD may cause lung injury by altering metabolism, immunity, fibrosis, and apoptosis in mouse offsprings. By better understanding the sex-specific gene-diet interactions behind maternal HSD-induced lung programming, our results could help develop effective personalized reprogramming strategies to prevent lung-related injuries.

## Supplementary Material



## Data Availability

The raw data have been deposited into sequence read archive (SRA) database: https://www.ncbi.nlm.nih.gov/bioproject/PRJNA977093.

## Appendix. Supplementary materials

Supplementary data including Supplementary Tables 1–20 and Supplementary Figs. 1–2 to this article can be found online at: http://dx.doi.org/10.29219/fnr.v68.11035.
