# Supplementary material for: Maternal nutrition and offspring lung health: sex-specific pathway modulation in fibrosis, metabolism, and immunity
Source: Food Nutr Res. 2025 Jan 3;69:10.29219/fnr.v69.11035. doi: 10.29219/fnr.v69.11035 (PMC11708518; doi:10.29219/fnr.v69.11035)
Supplement: Supplementary file 1 [file FNR-69-11035-s1.pdf]

Maternal Nutrition and Offspring Lung Health: Sex-Specific Pathway Modulation in Fibrosis, Metabolism, and Immunity

Shuangyi Zhao[1], Zhimin Chen[1], Huina Liu[1], Xinyan Wang[1], Xiuru Zhang[3], Huirong Shi[2,*]

[1]Department of Obstetrics, The First Affiliated Hospital of Zhengzhou University, Zhengzhou, 450052, China

[2]Department of Gynaecology, The First Affiliated Hospital of Zhengzhou University, Zhengzhou, 450052, China

[3]Department of Surgery of Spine and Spinal Cord, Henan Provincial People's Hospital, Zhengzhou, 450003, China

***Corresponding author**

Huirong Shi

Department of Gynaecology, The First Affiliated Hospital of Zhengzhou University, Zhengzhou, China (450052), Email address: hrshi2011@163.com

Xiuru Zhang

Department of Surgery of Spine and Spinal Cord, Henan Provincial People's Hospital, Zhengzhou, China (450003), Email address: zhangxiuru@zzu.edu.cn

Table S1. Composition of the standard diet AIN-93G

	gm%	kcal%
Protein	20.3	20.3
Carbohydrate	63.95	63.95
Fat	7	15.75
Total		100
kcal/gm	4	
**Ingredient**	**gm**	**kcal**
Casein	200	800
L-Cystine	3	12
Corn Starch	397.5	1590
Maltodextrin	132	528
Sucrose	100	400
Cellulose	50	0
Soybean Oil	70	630
Vitamin Mix V10037	10	40
Mineral Mix S10022G	35	0
Choline Bitartrate	2.5	0
Total	1000	3850

Table S2. The up-regulated and down-regulated DEGs of lung in VMOM and HSDMOM.

Gene ID	Gene Symbol	Qvalue (HSDMOM / VMOM)	up/down
100034684	Cstdc5	0.038502579	up
100039053	Gm2023	4.26E-06	up
100041599	LOC100041599	0.001629297	up
100041735	Gm3488	0.015148024	up
100503085	Klhl3	5.81E-05	up
101056232	Gm29804	0.035634996	up
104183	Chil4	7.39E-04	up
104816	Aspg	0.041823934	up
105244006	Gm39701	0.046320047	up
105244999	Gm40514	7.55E-04	up
105246825	Gm42049	9.45E-04	up
105247234	Gm42368	0.008839258	up
107239	Carns1	9.42E-06	up
107934	Celsr3	0.021387142	up
108169013	Gm46894	6.23E-05	up
110094	Phka2	7.86E-09	up
110595	Timp4	0.001574055	up
110749	Chaf1b	0.015365265	up
11434	Acr	0.004573431	up
11488	Adam11	0.020055837	up
115487803	Gm51918	1.99E-07	up
115488029	LOC115488029	0.006310278	up
115488503	Gm52197	3.08E-06	up
11685	Alox12e	0.039508099	up
11847	Arg2	0.041781646	up
12144	Blm	0.022237755	up
12338	Capn6	0.029412881	up
12659	Ovgp1	0.019374603	up
12696	Cirbp	2.22E-08	up
12740	Cldn4	0.029918265	up
12789	Cnga2	0.049319293	up
12794	Cnih2	0.010882495	up
12985	Csf3	0.034351009	up
13116	Cyp46a1	0.003332478	up
13717	Eln	0.001266398	up
14187	Akr1b8	0.002569105	up
14221	Fjx1	3.92E-06	up
14599	Gh	0.02773231	up
15360	Hmgcs2	4.75E-04	up
16178	Il1r2	0.02075113	up
16365	Acod1	0.009161802	up
16483	Kap	0.045948896	up
16625	Serpina3c	0.002420821	up
17523	Mpo	0.025895871	up
17831	Muc2	0.025966931	up
18302	Oit3	0.013162164	up
18405	Orm1	0.017598405	up
18574	Pde1b	0.01085072	up
18616	Peg3	7.38E-05	up
195209	Zfp469	4.38E-07	up
19737	Rgs5	2.47E-18	up
20210	Saa3	0.007094574	up
20310	Cxcl2	0.034351009	up
20528	Slc2a4	0.02711247	up

207911	Mchr1	0.019832875	up
20862	Stfa2	0.007420031	up
209540	Rtl9	0.00347697	up
209966	Pgbd5	0.034872386	up
211187	Lrtm2	0.006255876	up
213002	Ifitm6	0.028060513	up
213417	Klhdc8a	0.008761495	up
213980	Fbxw10	0.008843525	up
213989	Tmem82	0.008775735	up
214230	Pak6	0.00364353	up
215446	Entpd3	0.024190948	up
216961	Coro6	5.41E-06	up
218203	Mylip	6.81E-10	up
22236	Ugt1a2	0.002925543	up
227615	Tmem203	0.002546926	up
227632	Kcnt1	2.37E-06	up
22776	Zim1	0.015330621	up
230810	Slc30a2	0.031570053	up
231830	Micall2	0.001760808	up
233908	Fus	3.41E-05	up
234684	Lrrc29	0.013231696	up
243078	Tecrl	0.013043056	up
245195	Retnlg	0.04392461	up
258783	Olfr920	0.001629297	up
268885	Stfa2l1	0.034245469	up
268958	Capn11	0.026084625	up
276952	Rasl10b	0.01773804	up
319582	Trmt9b	0.015365265	up
320563	Islr2	0.024472326	up
381404	Pabpc1l	9.95E-04	up
381813	Prmt8	0.001436695	up
381823	Apold1	0.003879888	up
384071	Slc25a34	1.99E-07	up
433016	Cstdc4	0.038416062	up
433470	AA467197	0.032413254	up
494468	Armcx5	0.00269855	up
50501	Prok2	0.00240875	up
50701	Elane	0.029515537	up
50934	Slc7a8	0.046946075	up
53417	Hif3a	1.64E-04	up
546644	Ly6g	0.030816174	up
56461	Kcnip3	0.004715815	up
56753	Tacstd2	0.034245469	up
57262	Retnla	0.049429168	up
64177	Trpv6	1.95E-05	up
68039	Nmb	0.027011697	up
69655	Cd164l2	0.030510353	up
70835	Prss22	0.029515537	up
70873	Cnbd2	0.001486203	up
71838	Phf7	1.56E-04	up
73988	4930438A08Rik	0.027011697	up
74080	Nmnat3	0.031755347	up
74281	Spatc1	0.036273855	up
74337	Palm3	0.031914987	up
77974	Rdh12	0.034140014	up
78751	Zc3h6	1.88E-06	up
85031	Pla1a	0.043760232	up
93710	Pcdhga2	0.026081063	up

100042493	Ccl21b	2.24E-07	down
100043125	Cd300ld5	6.97E-05	down
100043497	Nat8f7	0.004805311	down
100862324	LOC100862324	0.043671255	down
102638882	Gm35339	0.021069744	down
109700	Itga1	0.014457244	down
115489122	Gm52433	6.88E-04	down
11567	Avil	0.043861927	down
12062	Bdkrb2	2.44E-04	down
121022	Mrps6	0.010066789	down
12301	Cacybp	4.02E-14	down
12304	Pdia4	1.23E-06	down
12317	Calr	1.95E-05	down
12350	Car3	1.05E-08	down
12406	Serpinh1	2.44E-04	down
12443	Ccnd1	0.001629297	down
12879	Cys1	0.001275529	down
13124	Cyp8b1	0.031408656	down
13363	Dhh	0.004924414	down
13586	Ear1	0.001986259	down
14080	Fabp1	0.015354103	down
14228	Fkbp4	6.59E-07	down
14311	Cidec	0.001266398	down
14419	Gal	0.021151217	down
14828	Hspa5	5.41E-05	down
15481	Hspa8	1.14E-27	down
15502	Dnaja1	6.11E-11	down
15505	Hsph1	7.25E-35	down
15507	Hspb1	4.14E-05	down
15510	Hspd1	5.60E-10	down
15511	Hspa1b	2.58E-21	down
15516	Hsp90ab1	3.36E-16	down
15519	Hsp90aa1	4.04E-16	down
15528	Hspe1	2.87E-06	down
16639	Klra8	0.01200491	down
170786	Cd209a	6.68E-06	down
171166	Mcoln3	7.37E-04	down
18124	Nr4a3	0.011111572	down
18218	Dusp8	2.32E-06	down
18415	Hspa4l	3.23E-14	down
18451	P4ha1	9.78E-12	down
19220	Ptgfr	0.044797973	down
193740	Hspa1a	1.16E-06	down
20867	Stip1	2.84E-15	down
212448	9330159F19Rik	0.012699372	down
212706	N4bp3	1.20E-05	down
214639	4930486L24Rik	0.006170123	down
217737	Ahsa1	3.89E-13	down
219132	Phf11d	0.002031658	down
22027	Hsp90b1	5.31E-10	down
231842	Amz1	0.011319953	down
243312	Elfn1	0.034245469	down
243655	Klre1	0.001527706	down
244723	Olfm2	7.00E-08	down
246133	Kcne2	4.72E-04	down
26944	Tinag	0.035289753	down
269643	Ppp2r2c	5.48E-04	down
286940	Flnb	5.41E-06	down

319162	H2aw	0.011111572	down
320265	Tafa1	0.043257293	down
328561	Apol10b	0.048655	down
53325	Banp	1.09E-08	down
53881	Slc5a3	2.02E-07	down
56455	Dynll1	0.010109662	down
58233	Dnaja4	4.87E-05	down
63954	Rbp7	7.64E-04	down
66895	Pxdc1	1.49E-08	down
66917	Chordc1	4.29E-09	down
67434	Ankrd33b	5.92E-20	down
67878	Tmem33	1.36E-06	down
68310	Zmym1	4.63E-08	down
70356	St13	3.92E-06	down
72630	Hspa12b	1.50E-08	down
73710	Tubb2b	0.001196874	down
74499	Sost	0.011607814	down
76737	Creld2	6.86E-06	down
76933	Ifi27l2a	9.84E-07	down
80891	Fcrls	0.003963209	down
81489	Dnajb1	1.51E-05	down
97114	H3c15	0.029180648	down
99899	Ifi44	0.002192569	down

Table S3. The up-regulated and down-regulated DEGs of lung in VFOM and HSDFOM.

Gene ID	Gene Symbol	Qvalue (HSDFOM / VFOM)	up/down
100034251	Wfdc17	0.001942094	up
100034684	Cstdc5	0.005530671	up
100039484	Gm2260	6.04E-07	up
100039503	Gm2274	6.04E-07	up
100039939	Gm2506	0.006020662	up
100042493	Ccl21b	5.01E-05	up
100502825	Rpl37rt	7.74E-04	up
100503583	Fsbp	0.003853965	up
101056241	Gm29808	0.008370888	up
103784	Wdr92	1.02E-14	up
108168679	LOC108168679	1.03E-06	up
108169010	LOC108169010	0.003805483	up
13653	Egr1	0.031648958	up
14281	Fos	0.008675052	up
14373	G0s2	4.71E-15	up
14813	Grin2c	2.30E-04	up
16007	Ccn1	0.006164418	up
16855	Lgals4	0.008481405	up
17394	Mmp8	1.91E-04	up
17884	Myh4	1.02E-05	up
17996	Neb	0.041579993	up
18616	Peg3	0.003038033	up
19225	Ptgs2	0.001596983	up
19252	Dusp1	0.002416431	up
19737	Rgs5	6.25E-05	up
20201	S100a8	0.012576329	up
20202	S100a9	0.02503842	up
20210	Saa3	0.003258602	up
20311	Cxcl5	0.0191997	up
20862	Stfa2	0.039649947	up
213236	Dnd1	0.03337162	up
215446	Entpd3	7.04E-04	up
21906	Otop1	1.04E-04	up
21925	Tnnc2	0.012197601	up
22227	Ucp1	0.012197601	up
227522	Rpp38	2.43E-05	up
227632	Kcnt1	0.012197601	up
231724	Rad9b	0.018357716	up
241275	Noxa1	0.012238884	up
245126	Tarm1	0.015052331	up
245195	Retnlg	0.017780844	up
268885	Stfa2l1	0.009193051	up
268932	Caskin1	0.020711968	up
268958	Capn11	2.03E-04	up
330723	Htra4	0.016733692	up
353235	Pcdha8	0.034340546	up
381404	Pabpc1l	0.036370133	up
433016	Cstdc4	0.008305546	up
50501	Prok2	0.00789189	up
53422	Ybx2	0.002245524	up
54524	Syt6	0.013104362	up
57814	Kcne4	0.028884457	up
633057	Gm7102	0.01807092	up

67855	Asprv1	0.002660326	up
68468	Ly6g6c	0.002596684	up
68891	Cd177	4.88E-05	up
74843	Mss51	0.005813758	up
75697	C2cd4b	5.65E-04	up
93709	Pcdhga1	1.10E-05	up
100034361	Mfap1b	0.014290946	down
100038882	Isg15	7.91E-06	down
100039192	Tmem254c	0.047005555	down
100039796	Tgtp2	1.54E-13	down
100041504	LOC100041504	1.09E-08	down
100041546	Ly6c2	0.001134948	down
100042856	Gm4070	2.10E-28	down
100702	Gbp6	2.67E-24	down
102639105	Gm35498	0.014390968	down
102639543	Ifi206	2.89E-08	down
108078	Olr1	0.012197601	down
108167533	Gm46041	1.39E-04	down
110558	H2-Q9	0.0014798	down
11287	Pzp	0.042291396	down
115487832	Gm51936	5.76E-04	down
11657	Alb	0.040340618	down
116847	Prelp	0.005687309	down
11801	Cd5l	1.06E-05	down
12010	B2m	1.12E-16	down
12265	Ciita	8.02E-04	down
12514	Cd68	0.015052331	down
12769	Ccr9	0.047545896	down
12816	Col12a1	0.048354304	down
13170	Dbp	0.01770775	down
13521	Slc26a2	4.39E-05	down
14080	Fabp1	8.46E-05	down
14276	Folr2	0.021962675	down
14366	Fzd4	0.004172529	down
14469	Gbp2	4.63E-16	down
14473	Gc	0.014290946	down
14964	H2-D1	1.12E-07	down
15018	H2-Q7	1.71E-07	down
15234	Hgf	0.004301641	down
15930	Ido1	0.013437729	down
15944	Irgm1	1.59E-10	down
15951	Ifi204	0.001483398	down
15953	Ifi47	9.21E-12	down
15957	Ifit1	1.75E-06	down
15958	Ifit2	4.12E-09	down
15959	Ifit3	0.029683929	down
16145	Igtp	1.70E-25	down
16160	Il12b	0.029518852	down
16194	Il6ra	0.006271652	down
16391	Irf9	1.26E-05	down
16411	Itgax	0.003109491	down
16551	Kif11	0.021980187	down
16599	Klf3	1.63E-07	down
16640	Klra9	0.045752964	down
16913	Psmb8	1.87E-06	down
170638	Hpcal4	0.044252863	down
170745	Xpnpep2	0.008038296	down
17086	Ncr1	0.014390968	down

17122	Mxd4	2.04E-04	down
17167	Marco	0.045351575	down
17345	Mki67	0.031502285	down
17472	Gbp4	6.55E-22	down
17886	Myh9	0.004301641	down
17948	Naip2	0.00995943	down
18646	Prf1	0.008273515	down
18795	Plcb1	0.001094664	down
19039	Lgals3bp	7.14E-07	down
19153	Prx	1.77E-06	down
19274	Ptprm	1.09E-08	down
195564	Skint3	0.046525095	down
20128	Trim30a	5.38E-05	down
20299	Ccl22	0.005087822	down
20612	Siglec1	7.05E-05	down
20846	Stat1	6.56E-09	down
20847	Stat2	1.01E-05	down
20877	Aurkb	0.047345642	down
20893	Bhlhe40	0.001130457	down
209200	Dtx3l	3.95E-06	down
209387	Trim30d	7.76E-05	down
20970	Sdc3	3.30E-04	down
210356	Nckap5	2.32E-06	down
21354	Tap1	1.70E-25	down
21679	Tead4	1.85E-05	down
217166	Nr1d1	1.45E-04	down
21822	Tgtp1	1.69E-28	down
21847	Klf10	7.05E-05	down
219131	Phf11a	0.017780844	down
219132	Phf11d	3.53E-06	down
21973	Top2a	0.030781693	down
22139	Ttr	5.38E-05	down
22169	Cmpk2	3.49E-04	down
22262	Uox	0.048354304	down
229898	Gbp5	2.17E-10	down
229900	Gbp7	2.42E-15	down
230316	Megf9	0.048354304	down
231507	Plac8	0.044471696	down
232801	Lilra5	0.04011584	down
234258	Neil3	0.047274531	down
234311	Ddx60	0.017086959	down
234673	Ces2e	0.020179158	down
236451	Phf11b	3.87E-04	down
238393	Serpina3f	0.006264569	down
238803	Zfp366	1.29E-04	down
239528	Ago2	0.001263115	down
23960	Oas1g	7.75E-06	down
23962	Oasl2	2.55E-06	down
240327	Gm4951	1.32E-21	down
24110	Usp18	5.06E-04	down
241327	Olfml2a	1.68E-04	down
244281	Myo16	0.034161666	down
244723	Olfm2	0.011465415	down
245240	9930111J21Rik2	0.030115915	down
246256	Fcgr4	0.038929705	down
246727	Oas3	4.12E-09	down
246728	Oas2	0.025666515	down
246730	Oas1a	7.27E-11	down

269643	Ppp2r2c	0.046043588	down
27400	Hsd17b6	0.041404553	down
27413	Abcb11	0.042164547	down
276950	Slfn8	9.27E-04	down
320799	Zhx3	0.006156667	down
327959	Xaf1	4.47E-06	down
353237	Pcdhac2	0.003561658	down
382053	Ces3a	0.016013057	down
384309	Trim56	7.79E-06	down
432555	Gm5431	4.10E-04	down
434325	Tmem221	8.62E-04	down
434341	Nlrc5	2.09E-37	down
442834	D830031N03Rik	0.022014152	down
52855	Lair1	0.01127368	down
54123	Irf7	2.20E-08	down
54396	Irgm2	3.50E-13	down
54608	Abhd2	9.04E-04	down
547253	Parp14	9.23E-10	down
55932	Gbp3	1.53E-13	down
56620	Clec4n	5.58E-04	down
57248	Ly6i	1.32E-04	down
58185	Rsad2	0.01893252	down
58203	Zbp1	5.68E-11	down
60440	Iigp1	3.64E-10	down
60533	Cd274	3.23E-07	down
619547	Rpl34-ps1	0.030781693	down
620913	Gm12185	2.09E-04	down
621823	Psme2b	0.037326126	down
626578	Gbp10	8.43E-16	down
631323	Gm12250	1.15E-11	down
65221	Slc15a3	0.007515707	down
667214	9930111J21Rik1	4.71E-07	down
667370	Ifit3b	0.026829411	down
66929	Asf1b	0.007981936	down
67138	Herc6	1.53E-05	down
672511	Rnf213	3.01E-07	down
67775	Rtp4	6.58E-06	down
68817	Ddi2	5.92E-04	down
69065	Chac1	0.019080822	down
69550	Bst2	2.72E-05	down
71586	Ifih1	4.87E-06	down
71898	Apol9b	5.42E-04	down
73340	Nptxr	0.038971055	down
74481	Batf2	5.11E-11	down
74558	Gvin1	8.88E-08	down
75345	Slamf7	0.02958007	down
76933	Ifi27l2a	1.73E-13	down
78943	Ern1	0.014216851	down
79362	Bhlhe41	0.030075826	down
80861	Dhx58	7.05E-05	down
80909	Castor2	0.032312063	down
96875	Prg4	0.02511177	down
98999	Znfx1	4.31E-07	down
99899	Ifi44	4.45E-07	down

Table S4. The up-regulated and down-regulated DEGs of lung in VMOM and VFOM.

Gene ID	Gene Symbol	Qvalue (VMOM / VFOM)	up/down
100042493	Ccl21b	2.48E-06	up
100861598	Gm21064	0.044267937	up
100862261	Fam205a3	0.02030973	up
12609	Cebpd	1.28E-06	up
14219	Ccn2	2.37E-04	up
14605	Tsc22d3	0.003228048	up
15505	Hsph1	6.81E-10	up
15507	Hspb1	3.89E-10	up
15511	Hspa1b	3.74E-04	up
19252	Dusp1	7.58E-12	up
20592	Kdm5d	1.41E-16	up
22290	Uty	5.02E-52	up
235320	Zbtb16	6.39E-04	up
26897	Acot1	1.78E-04	up
26900	Ddx3y	1.39E-48	up
26908	Eif2s3y	4.71E-83	up
27273	Pdk4	0.014231909	up
57875	Angptl4	0.021633069	up
666329	Gm3317	0.004460683	up
72287	Plekhf1	0.044267937	up
74747	Ddit4	7.01E-04	up
100039192	Tmem254c	0.046505779	down
12116	Bhmt	0.021633069	down
13096	Cyp2c37	0.014231909	down
15951	Ifi204	0.001780521	down
16483	Kap	5.48E-04	down
17079	Cd180	0.047144234	down
17906	Myl2	7.47E-04	down
20558	Slfn4	0.00213481	down
21822	Tgtp1	5.20E-06	down
230163	Aldob	0.002283095	down
394436	Ugt1a1	0.006359011	down
50708	H1f2	0.02002335	down
57248	Ly6i	0.04956327	down
58203	Zbp1	4.40E-04	down
60533	Cd274	2.37E-04	down
626578	Gbp10	0.045125435	down
76279	Cyp2d26	0.034707028	down

Table S5. KEGG pathways enriched in higher expression genes unique to VMOM.

KEGG Pa	KEGG Pathway Term Desc	KEGG Pathway Term	KEGG Path	Term Can	Total Can	Rich Rati	P value	Q value
4330	Notch signaling pathway	Environmental Inform	Signal trans	5	229	0.09259	1.48E-02	0.80508
5202	Transcriptional misregulation in cancer	Human Diseases	Cancer: ove	10	229	0.05587	2.29E-02	0.80508
5217	Basal cell carcinoma	Human Diseases	Cancer: spe	5	229	0.07937	2.71E-02	0.80508
5323	Rheumatoid arthritis	Human Diseases	Immune dise	6	229	0.06977	2.83E-02	0.80508
5031	Amphetamine addiction	Human Diseases	Substance c	5	229	0.07246	3.80E-02	0.80508
5140	Leishmaniasis	Human Diseases	Infectious di	5	229	0.07246	3.80E-02	0.80508
5221	Acute myeloid leukemia	Human Diseases	Cancer: spe	5	229	0.07143	4.01E-02	0.80508
5224	Breast cancer	Human Diseases	Cancer: spe	8	229	0.05442	4.52E-02	0.80508
600	Sphingolipid metabolism	Metabolism	Lipid metabo	5	229	0.10417	9.10E-03	0.80508
520	Amino sugar and nucleotide sugar metabolism	Metabolism	Carbohydrat	5	229	0.10204	9.92E-03	0.80508
511	Other glycan degradation	Metabolism	Glycan biosy	3	229	0.16667	1.17E-02	0.80508
4380	Osteoclast differentiation	Organismal Systems	Developmer	9	229	0.07258	6.24E-03	0.80508
4916	Melanogenesis	Organismal Systems	Endocrine s	7	229	0.07	1.82E-02	0.80508

Table S6. KEGG pathways enriched in higher expression genes shared in VMOM and HSDMOM.

KEGG Pa	KEGG Pathway Term Desc	KEGG Pa	KEGG Pa	Term Can	Total Can	Term Ger	Total Ger	Rich Ratio	P value	Q value
4110	Cell cycle	Cellular Pr	Cell growt	72	3153	123	8491	0.58537	9.93E-07	6.85E-05
4070	Phosphatidylinositol signaling system	Environme	Signal trar	58	3153	98	8491	0.59184	6.89E-06	2.97E-04
4064	NF-kappa B signaling pathway	Environme	Signal trar	62	3153	108	8491	0.57407	1.32E-05	4.55E-04
4310	Wnt signaling pathway	Environme	Signal trar	85	3153	161	8491	0.52795	3.18E-05	8.44E-04
3030	DNA replication	Genetic In	Replicatio	30	3153	35	8491	0.85714	4.06E-09	7.00E-07
970	Aminoacyl-tRNA biosynthesis	Genetic In	Translatio	35	3153	44	8491	0.79545	1.02E-08	1.17E-06
3420	Nucleotide excision repair	Genetic In	Replicatio	32	3153	43	8491	0.74419	6.91E-07	5.96E-05
3008	Ribosome biogenesis in eukaryotes	Genetic In	Translatio	50	3153	79	8491	0.63291	1.95E-06	1.12E-04
3430	Mismatch repair	Genetic In	Replicatio	19	3153	22	8491	0.86364	2.72E-06	1.34E-04
4120	Ubiquitin mediated proteolysis	Genetic In	Folding, s	76	3153	138	8491	0.55072	1.18E-05	4.54E-04
3460	Fanconi anemia pathway	Genetic In	Replicatio	34	3153	51	8491	0.66667	1.70E-05	5.34E-04
3450	Non-homologous end-joining	Genetic In	Replicatio	12	3153	13	8491	0.92308	5.80E-05	0.00143
3410	Base excision repair	Genetic In	Replicatio	24	3153	34	8491	0.70588	7.49E-05	0.00172
3022	Basal transcription factors	Genetic In	Transcript	28	3153	44	8491	0.63636	3.14E-04	0.00515
3018	RNA degradation	Genetic In	Folding, s	46	3153	82	8491	0.56098	3.47E-04	0.00545
5168	Herpes simplex virus 1 infection	Human Di	Infectious	258	3153	432	8491	0.59722	1.28E-22	4.41E-20
5225	Hepatocellular carcinoma	Human Di	Cancer: s	85	3153	171	8491	0.49708	4.69E-04	0.00647
5340	Primary immunodeficiency	Human Di	Immune d	23	3153	36	8491	0.63889	9.91E-04	0.01221
450	Selenocompound metabolism	Metabolisr	Metabolisr	15	3153	17	8491	0.88235	2.00E-05	5.75E-04
240	Pyrimidine metabolism	Metabolisr	Nucleotide	36	3153	58	8491	0.62069	9.63E-05	0.00208
230	Purine metabolism	Metabolisr	Nucleotide	72	3153	136	8491	0.52941	1.11E-04	0.00226
562	Inositol phosphate metabolism	Metabolisr	Carbohydr	43	3153	73	8491	0.58904	1.21E-04	0.00233
670	One carbon pool by folate	Metabolisr	Metabolisr	15	3153	19	8491	0.78947	2.44E-04	0.00444
900	Terpenoid backbone biosynthesis	Metabolisr	Metabolisr	17	3153	23	8491	0.73913	3.66E-04	0.00549
310	Lysine degradation	Metabolisr	Amino aci	36	3153	61	8491	0.59016	4.05E-04	0.00582
520	Amino sugar and nucleotide sugar metaboli	Metabolisr	Carbohydr	30	3153	49	8491	0.61224	5.11E-04	0.00678
4914	Progesterone-mediated oocyte maturation	Organisma	Endocrine	50	3153	90	8491	0.55556	2.69E-04	0.00464
4360	Axon guidance	Organisma	Developm	88	3153	180	8491	0.48889	7.45E-04	0.00952

Table S7. KEGG pathways enriched in higher expression genes unique to HSDMOM.

KEGG Pa	KEGG Pathway Term Desc	KEGG Pa	KEGG Pa	Term Can	Total Can	Term Ger	Total Ger	Rich Ratio	P value	Q value
4060	Cytokine-cytokine receptor interaction	Environme	Signaling	25	241	295	8491	0.08475	8.76E-07	2.46E-04
4061	Viral protein interaction with cytokine and c	Environme	Signaling	13	241	102	8491	0.12745	5.57E-06	7.82E-04
4668	TNF signaling pathway	Environme	Signal trar	9	241	113	8491	0.07965	4.73E-03	0.16952
4151	PI3K-Akt signaling pathway	Environme	Signal trar	19	241	355	8491	0.05352	5.80E-03	0.16952
4630	Jak-STAT signaling pathway	Environme	Signal trar	11	241	165	8491	0.06667	7.24E-03	0.16952
5144	Malaria	Human Di	Infectious	9	241	56	8491	0.16071	2.43E-05	0.00228
5323	Rheumatoid arthritis	Human Di	Immune d	9	241	86	8491	0.10465	7.06E-04	0.04956
5143	African trypanosomiasis	Human Di	Infectious	5	241	38	8491	0.13158	4.13E-03	0.16952
5134	Legionellosis	Human Di	Infectious	6	241	59	8491	0.10169	6.30E-03	0.16952
5164	Influenza A	Human Di	Infectious	11	241	163	8491	0.06748	6.62E-03	0.16952
5162	Measles	Human Di	Infectious	10	241	142	8491	0.07042	7.07E-03	0.16952
5142	Chagas disease (American trypanosomias	Human Di	Infectious	8	241	102	8491	0.07843	8.30E-03	0.17937
4962	Vasopressin-regulated water reabsorption	Organisma	Excretory	6	241	43	8491	0.13953	1.24E-03	0.06977

Table S8. KEGG pathways enriched in higher expression genes unique to VFOM.

KEGG Pa	KEGG Pathway Term Desc	KEGG Pa	KEGG Pa	Term Can	Total Can	Term Ger	Total Gen	Rich Rati	P value	Q value
4115	p53 signaling pathway	Cellular Pr	Cell growt	10	256	71	8491	0.14085	4.76E-05	0.00467
5204	Chemical carcinogenesis	Human Di	Cancer: ov	11	256	95	8491	0.11579	1.25E-04	0.00757
5143	African trypanosomiasis	Human Di	Infectious	6	256	38	8491	0.15789	8.69E-04	0.03749
5144	Malaria	Human Di	Infectious	6	256	56	8491	0.10714	6.51E-03	0.17871
591	Linoleic acid metabolism	Metabolisr	Lipid meta	10	256	49	8491	0.20408	1.52E-06	4.46E-04
830	Retinol metabolism	Metabolisr	Metabolisr	13	256	91	8491	0.14286	2.96E-06	4.46E-04
140	Steroid hormone biosynthesis	Metabolisr	Lipid meta	11	256	88	8491	0.125	6.19E-05	0.00467
590	Arachidonic acid metabolism	Metabolisr	Lipid meta	10	256	88	8491	0.11364	2.97E-04	0.01493
53	Ascorbate and aldarate metabolism	Metabolisr	Carbohydr	5	256	27	8491	0.18519	1.12E-03	0.04236
310	Lysine degradation	Metabolisr	Amino aci	7	256	61	8491	0.11475	2.27E-03	0.07608
380	Tryptophan metabolism	Metabolisr	Amino aci	6	256	48	8491	0.125	3.00E-03	0.09074
4620	Toll-like receptor signaling pathway	Organisma	Immune s	8	256	98	8491	0.08163	9.30E-03	0.23403

Table S9. KEGG pathways enriched in higher expression genes shared in VFOM and HSDFOM.

KEGG Pa	KEGG Pathway Term Desc	KEGG Pa	KEGG Pa	Term Can	Total Can	Term Ger	Total Ger	Rich Rati	P value	Q value
4110	Cell cycle	Cellular Pı	Cell growt	73	3196	123	8491	0.5935	7.26E-07	6.26E-05
4140	Autophagy - animal	Cellular Pı	Transport	71	3196	138	8491	0.51449	6.00E-04	0.00668
4070	Phosphatidylinositol signaling syste	Environme	Signal trar	60	3196	98	8491	0.61224	1.66E-06	9.57E-05
4310	Wnt signaling pathway	Environme	Signal trar	84	3196	161	8491	0.52174	1.07E-04	0.00217
4064	NF-kappa B signaling pathway	Environme	Signal trar	60	3196	108	8491	0.55556	1.07E-04	0.00217
4152	AMPK signaling pathway	Environme	Signal trar	68	3196	126	8491	0.53968	1.28E-04	0.00245
4150	mTOR signaling pathway	Environme	Signal trar	78	3196	153	8491	0.5098	4.76E-04	0.00587
3030	DNA replication	Genetic In	Replicatio	29	3196	35	8491	0.82857	5.03E-08	8.67E-06
970	Aminoacyl-tRNA biosynthesis	Genetic In	Translatio	34	3196	44	8491	0.77273	9.19E-08	1.06E-05
3420	Nucleotide excision repair	Genetic In	Replicatio	32	3196	43	8491	0.74419	9.83E-07	6.78E-05
3008	Ribosome biogenesis in eukaryotes	Genetic In	Translatio	50	3196	79	8491	0.63291	3.08E-06	1.52E-04
3430	Mismatch repair	Genetic In	Replicatio	18	3196	22	8491	0.81818	2.83E-05	0.00109
4120	Ubiquitin mediated proteolysis	Genetic In	Folding, so	75	3196	138	8491	0.54348	4.28E-05	0.00148
3450	Non-homologous end-joining	Genetic In	Replicatio	12	3196	13	8491	0.92308	6.78E-05	0.00182
3460	Fanconi anemia pathway	Genetic In	Replicatio	33	3196	51	8491	0.64706	7.76E-05	0.00191
3410	Base excision repair	Genetic In	Replicatio	24	3196	34	8491	0.70588	9.63E-05	0.00217
3022	Basal transcription factors	Genetic In	Transcript	28	3196	44	8491	0.63636	4.07E-04	0.00524
5168	Herpes simplex virus 1 infection	Human Di	Infectious	266	3196	432	8491	0.61574	4.13E-25	1.43E-22
5225	Hepatocellular carcinoma	Human Di	Cancer: sp	88	3196	171	8491	0.51462	1.40E-04	0.00254
5217	Basal cell carcinoma	Human Di	Cancer: sp	38	3196	63	8491	0.60317	2.07E-04	0.00333
4931	Insulin resistance	Human Di	Endocrine	60	3196	110	8491	0.54545	2.12E-04	0.00333
1523	Antifolate resistance	Human Di	Drug resis	21	3196	30	8491	0.7	3.20E-04	0.00442
5200	Pathways in cancer	Human Di	Cancer: ov	239	3196	538	8491	0.44424	5.15E-04	0.00613
5210	Colorectal cancer	Human Di	Cancer: sp	48	3196	88	8491	0.54545	8.81E-04	0.00894
450	Selenocompound metabolism	Metabolisr	Metabolisr	15	3196	17	8491	0.88235	2.42E-05	0.00104
562	Inositol phosphate metabolism	Metabolisr	Carbohydr	44	3196	73	8491	0.60274	6.85E-05	0.00182
670	One carbon pool by folate	Metabolisr	Metabolisr	15	3196	19	8491	0.78947	2.91E-04	0.00418
130	Ubiquinone and other terpenoid-qu	Metabolisr	Metabolisr	10	3196	11	8491	0.90909	4.10E-04	0.00524
310	Lysine degradation	Metabolisr	Amino acio	36	3196	61	8491	0.59016	5.47E-04	0.00629
230	Purine metabolism	Metabolisr	Nucleotide	70	3196	136	8491	0.51471	6.44E-04	0.00694
51	Fructose and mannose metabolism	Metabolisr	Carbohydr	23	3196	35	8491	0.65714	6.88E-04	0.00719
240	Pyrimidine metabolism	Metabolisr	Nucleotide	34	3196	58	8491	0.58621	9.15E-04	0.00902
4380	Osteoclast differentiation	Organisma	Developm	68	3196	124	8491	0.54839	6.62E-05	0.00182
4914	Progesterone-mediated oocyte ma	Organisma	Endocrine	51	3196	90	8491	0.56667	1.77E-04	0.00306
4360	Axon guidance	Organisma	Developm	91	3196	180	8491	0.50556	2.44E-04	0.00367

Table S10. KEGG pathways enriched in higher expression genes unique to HSDFOM.

KEGG Pa	KEGG Pathway Term Desc	KEGG Pa	KEGG Pa	Term Can	Total Can	Term Ger	Total Gen	Rich Rati	P value	Q value
4514	Cell adhesion molecules (CAMs)	Environme	Signaling	10	202	169	8491	0.05917	7.00E-03	0.23884
4022	cGMP-PKG signaling pathway	Environme	Signal trar	10	202	173	8491	0.0578	8.20E-03	0.23884
5416	Viral myocarditis	Human Di	Cardiovas	8	202	86	8491	0.09302	9.67E-04	0.14448
4940	Type I diabetes mellitus	Human Di	Endocrine	7	202	68	8491	0.10294	1.10E-03	0.14448
5145	Toxoplasmosis	Human Di	Infectious	8	202	108	8491	0.07407	4.12E-03	0.23884
5323	Rheumatoid arthritis	Human Di	Immune d	7	202	86	8491	0.0814	4.27E-03	0.23884
5169	Epstein-Barr virus infection	Human Di	Infectious	12	202	226	8491	0.0531	7.64E-03	0.23884
5130	Pathogenic Escherichia coli infectio	Human Di	Infectious	11	202	205	8491	0.05366	9.72E-03	0.24346
603	Glycosphingolipid biosynthesis - glo	Metabolisr	Glycan bio	3	202	16	8491	0.1875	5.91E-03	0.23884
4612	Antigen processing and presentatio	Organisma	Immune s	7	202	88	8491	0.07955	4.85E-03	0.23884

Table S11. KEGG pathways enriched in higher expression genes unique to VMOM.

KEGG Pa	KEGG Pathway Term Desc	KEGG Pa	KEGG Pa	Term Can	Total Can	Term Ger	Total Ger	Rich Rati	P value	Q value	Remark
4146	Peroxisome	Cellular Pı	Transport	5	141	84	8491	0.05952	1.27E-02	0.66495	VMOM
4216	Ferroptosis	Cellular Pı	Cell growt	3	141	41	8491	0.07317	3.02E-02	0.8565	VMOM
4330	Notch signaling pathway	Environme	Signal trar	4	141	54	8491	0.07407	1.21E-02	0.66495	VMOM
5416	Viral myocarditis	Human Di	Cardiovas	5	141	86	8491	0.05814	1.40E-02	0.66495	VMOM
511	Other glycan degradation	Metabolisr	Glycan bic	3	141	18	8491	0.16667	3.05E-03	0.66495	VMOM
603	Glycosphingolipid biosynthesis - g	Metabolisr	Glycan bic	2	141	16	8491	0.125	2.82E-02	0.8565	VMOM
61	Fatty acid biosynthesis	Metabolisr	Lipid meta	2	141	19	8491	0.10526	3.89E-02	0.92163	VMOM
520	Amino sugar and nucleotide suga	Metabolisr	Carbohydı	3	141	49	8491	0.06122	4.74E-02	0.92163	VMOM
3320	PPAR signaling pathway	Organisma	Endocrine	5	141	87	8491	0.05747	1.46E-02	0.66495	VMOM
4623	Cytosolic DNA-sensing pathway	Organisma	Immune s	4	141	64	8491	0.0625	2.15E-02	0.81241	VMOM

Table S12. KEGG pathways enriched in higher expression genes shared in VMOM and VFOM.

KEGG Pa	KEGG Pathway Term Desc	KEGG Pa	KEGG Pa	Term Can	Total Can	Term Ger	Total Ger	Rich Rati	P value	Q value
4110	Cell cycle	Cellular Pr	Cell growt	73	3241	123	8491	0.5935	1.35E-06	6.97E-05
4070	Phosphatidylinositol signaling system	Environme	Signal trar	61	3241	98	8491	0.62245	1.05E-06	6.97E-05
4310	Wnt signaling pathway	Environme	Signal trar	88	3241	161	8491	0.54658	1.34E-05	4.65E-04
4064	NF-kappa B signaling pathway	Environme	Signal trar	63	3241	108	8491	0.58333	1.53E-05	4.83E-04
4152	AMPK signaling pathway	Environme	Signal trar	66	3241	126	8491	0.52381	7.60E-04	0.00924
4150	mTOR signaling pathway	Environme	Signal trar	78	3241	153	8491	0.5098	7.74E-04	0.00924
3030	DNA replication	Genetic In	Replicatio	31	3241	35	8491	0.88571	8.29E-10	1.43E-07
970	Aminoacyl-tRNA biosynthesis	Genetic In	Translatio	35	3241	44	8491	0.79545	2.33E-08	2.69E-06
3008	Ribosome biogenesis in eukaryotes	Genetic In	Translatio	53	3241	79	8491	0.67089	1.62E-07	1.40E-05
3420	Nucleotide excision repair	Genetic In	Replicatio	32	3241	43	8491	0.74419	1.41E-06	6.97E-05
3430	Mismatch repair	Genetic In	Replicatio	19	3241	22	8491	0.86364	4.39E-06	1.69E-04
4120	Ubiquitin mediated proteolysis	Genetic In	Folding, sc	77	3241	138	8491	0.55797	1.73E-05	5.00E-04
3410	Base excision repair	Genetic In	Replicatio	25	3241	34	8491	0.73529	2.94E-05	7.83E-04
3460	Fanconi anemia pathway	Genetic In	Replicatio	34	3241	51	8491	0.66667	3.35E-05	8.27E-04
3450	Non-homologous end-joining	Genetic In	Replicatio	12	3241	13	8491	0.92308	7.96E-05	0.00162
3018	RNA degradation	Genetic In	Folding, sc	47	3241	82	8491	0.57317	3.19E-04	0.0046
3022	Basal transcription factors	Genetic In	Transcript	28	3241	44	8491	0.63636	5.31E-04	0.00707
5168	Herpes simplex virus 1 infection	Human Di	Infectious	264	3241	432	8491	0.61111	3.57E-23	1.23E-20
5217	Basal cell carcinoma	Human Di	Cancer: sp	39	3241	63	8491	0.61905	1.09E-04	0.00199
5225	Hepatocellular carcinoma	Human Di	Cancer: sp	89	3241	171	8491	0.52047	1.35E-04	0.00234
1523	Antifolate resistance	Human Di	Drug resis	21	3241	30	8491	0.7	4.01E-04	0.00555
5200	Pathways in cancer	Human Di	Cancer: ov	240	3241	538	8491	0.4461	9.43E-04	0.01052
450	Selenocompound metabolism	Metabolisr	Metabolisr	16	3241	17	8491	0.94118	2.16E-06	9.36E-05
562	Inositol phosphate metabolism	Metabolisr	Carbohydr	45	3241	73	8491	0.61644	3.84E-05	8.86E-04
670	One carbon pool by folate	Metabolisr	Metabolisr	16	3241	19	8491	0.84211	5.10E-05	0.0011
520	Amino sugar and nucleotide sugar me	Metabolisr	Carbohydr	32	3241	49	8491	0.65306	1.04E-04	0.00199
230	Purine metabolism	Metabolisr	Nucleotide	73	3241	136	8491	0.53676	1.54E-04	0.00254
240	Pyrimidine metabolism	Metabolisr	Nucleotide	36	3241	58	8491	0.62069	1.85E-04	0.00291
310	Lysine degradation	Metabolisr	Amino aci	37	3241	61	8491	0.60656	2.96E-04	0.00445
4914	Progesterone-mediated oocyte matura	Organisma	Endocrine	50	3241	90	8491	0.55556	5.72E-04	0.00733
4380	Osteoclast differentiation	Organisma	Developm	65	3241	124	8491	0.52419	8.10E-04	0.00934

Table S13. KEGG pathways enriched in higher expression genes unique to VFOM.

KEGG Pa	KEGG Pathway Term Desc	KEGG Pa	KEGG Pa	Term Can	Total Can	Term Gen	Total Gen	Rich Rati	P value	Q value
4115	p53 signaling pathway	Cellular Pr	Cell growt	6	211	71	8491	0.08451	8.21E-03	0.15996
5204	Chemical carcinogenesis	Human Di	Cancer: o	11	211	95	8491	0.11579	2.16E-05	0.0012
5143	African trypanosomiasis	Human Di	Infectious	5	211	38	8491	0.13158	2.33E-03	0.06466
830	Retinol metabolism	Metabolisr	Metabolisr	15	211	91	8491	0.16484	5.36E-09	1.49E-06
140	Steroid hormone biosynthesis	Metabolisr	Lipid meta	14	211	88	8491	0.15909	2.83E-08	3.93E-06
591	Linoleic acid metabolism	Metabolisr	Lipid meta	9	211	49	8491	0.18367	2.64E-06	1.84E-04
380	Tryptophan metabolism	Metabolisr	Amino aci	7	211	48	8491	0.14583	1.64E-04	0.0076
590	Arachidonic acid metabolism	Metabolisr	Lipid meta	8	211	88	8491	0.09091	1.49E-03	0.04596
310	Lysine degradation	Metabolisr	Amino aci	6	211	61	8491	0.09836	3.90E-03	0.0962
53	Ascorbate and aldarate metabolism	Metabolisr	Carbohydr	4	211	27	8491	0.14815	4.15E-03	0.0962
524	Neomycin, kanamycin and gentamicin bios	Metabolisr	Biosynthes	2	211	5	8491	0.4	5.85E-03	0.12509
232	Caffeine metabolism	Metabolisr	Biosynthes	2	211	6	8491	0.33333	8.63E-03	0.15996
4610	Complement and coagulation cascades	Organisma	Immune s	13	211	93	8491	0.13978	4.29E-07	3.97E-05
4750	Inflammatory mediator regulation of TRP c	Organisma	Sensory s	11	211	127	8491	0.08661	3.07E-04	0.01218
3320	PPAR signaling pathway	Organisma	Endocrine	8	211	87	8491	0.09195	1.38E-03	0.04596

Table S14. Molecular functions of pulmonary fibrosis associated DEGs in male and female mouse offspring.

GO_F Ter	GO_F Term Desc	GO_F Ter	GO_F Ter	Term Can	Total Can	Term Ger	Total Ger	Rich Ratio	P value	Q value	Remark
GO:00051	integrin binding	molecular	binding	4	36	141	20317	0.02837	1.10E-04	0.00672	Male
GO:00055	collagen binding	molecular	binding	3	36	72	20317	0.04167	2.80E-04	0.00854	Male
GO:00484	platelet-derived growth factor bind	molecular	binding	2	36	12	20317	0.16667	1.99E-04	0.00729	Male
GO:00051	platelet-derived growth factor rec	molecular	binding	4	36	15	20317	0.26667	1.12E-08	2.04E-06	Male
GO:00708	growth factor receptor binding	molecular	binding	3	36	8	20317	0.375	2.84E-07	2.60E-05	Male
GO:00308	actin-dependent ATPase activity	molecular	catalytic a	2	36	26	20317	0.07692	9.66E-04	0.0141	Male
GO:01025	phospholipase A2 activity (consur	molecular	catalytic a	2	36	26	20317	0.07692	9.66E-04	0.0141	Male
GO:01025	phospholipase A2 activity consum	molecular	catalytic a	2	36	26	20317	0.07692	9.66E-04	0.0141	Male
GO:00080	growth factor activity	molecular	molecular	4	36	153	20317	0.02614	1.51E-04	0.00691	Male
GO:00150	coreceptor activity	molecular	molecular	2	36	26	20317	0.07692	9.66E-04	0.0141	Male
GO:00151	chloride transmembrane transpor	molecular	transporte	2	36	18	20317	0.11111	4.59E-04	0.01049	Male
GO:00151	bicarbonate transmembrane trans	molecular	transporte	2	36	16	20317	0.125	3.61E-04	0.00943	Male
GO:00051	platelet-derived growth factor rec	molecular	binding	3	34	15	20317	0.2	1.92E-06	3.36E-04	Female
GO:00019	fibronectin binding	molecular	binding	3	34	31	20317	0.09677	1.86E-05	0.00163	Female
GO:00708	growth factor receptor binding	molecular	binding	2	34	8	20317	0.25	7.56E-05	0.00348	Female
GO:00198	kinesin binding	molecular	binding	3	34	50	20317	0.06	7.95E-05	0.00348	Female
GO:00484	platelet-derived growth factor bind	molecular	binding	2	34	12	20317	0.16667	1.78E-04	0.00621	Female
GO:00055	collagen binding	molecular	binding	3	34	72	20317	0.04167	2.36E-04	0.00688	Female
GO:00971	scaffold protein binding	molecular	binding	3	34	77	20317	0.03896	2.88E-04	0.00719	Female
GO:00051	signaling receptor binding	molecular	binding	5	34	411	20317	0.01217	5.67E-04	0.01241	Female
GO:00051	integrin binding	molecular	binding	3	34	141	20317	0.02128	1.67E-03	0.02253	Female
GO:00318	P2Y1 nucleotide receptor binding	molecular	binding	1	34	1	20317	1	1.67E-03	0.02253	Female
GO:00080	growth factor activity	molecular	binding	3	34	153	20317	0.01961	2.11E-03	0.02468	Female
GO:00055	protein binding	molecular	binding	17	34	5245	20317	0.00324	2.12E-03	0.02468	Female
GO:00018	opsonin binding	molecular	binding	1	34	2	20317	0.5	3.34E-03	0.02787	Female
GO:00358	death effector domain binding	molecular	binding	1	34	3	20317	0.33333	5.01E-03	0.03655	Female
GO:00050	protein kinase C binding	molecular	binding	2	34	68	20317	0.02941	5.78E-03	0.04045	Female
GO:00316	beta-1 adrenergic receptor bindin	molecular	binding	1	34	4	20317	0.25	6.68E-03	0.04174	Female
GO:00971	neuroligin family protein binding	molecular	binding	1	34	4	20317	0.25	6.68E-03	0.04174	Female
GO:00199	C-X3-C chemokine binding	molecular	binding	1	34	5	20317	0.2	8.34E-03	0.04375	Female
GO:00050	platelet-derived growth factor alpl	molecular	catalytic a	1	34	1	20317	1	1.67E-03	0.02253	Female
GO:00973	NAD-dependent histone deacetyl	molecular	catalytic a	1	34	1	20317	1	1.67E-03	0.02253	Female
GO:00045	beta-fructofuranosidase activity	molecular	catalytic a	1	34	2	20317	0.5	3.34E-03	0.02787	Female
GO:00045	oligo-1,6-glucosidase activity	molecular	catalytic a	1	34	2	20317	0.5	3.34E-03	0.02787	Female
GO:00360	protein-succinyllysine desuccinyla	molecular	catalytic a	1	34	2	20317	0.5	3.34E-03	0.02787	Female
GO:00616	protein-glutaryllysine deglutarylas	molecular	catalytic a	1	34	2	20317	0.5	3.34E-03	0.02787	Female

GO:00080	phosphoric diester hydrolase acti	molecular	catalytic a	2	34	58	20317	0.03448	4.24E-03	0.03371	Female
GO:00040	arginase activity	molecular	catalytic a	1	34	3	20317	0.33333	5.01E-03	0.03655	Female
GO:00082	calcium- and calmodulin-respons	molecular	catalytic a	1	34	5	20317	0.2	8.34E-03	0.04375	Female
GO:00168	hydrolase activity, acting on carb	molecular	catalytic a	1	34	5	20317	0.2	8.34E-03	0.04375	Female
GO:00170	myosin phosphatase regulator ac	molecular	molecular	1	34	4	20317	0.25	6.68E-03	0.04174	Female
GO:00601	apelin receptor activity	molecular	molecular	1	34	1	20317	1	1.67E-03	0.02253	Female
GO:00365	prosaposin receptor activity	molecular	molecular	1	34	2	20317	0.5	3.34E-03	0.02787	Female
GO:00302	estrogen receptor activity	molecular	molecular	1	34	5	20317	0.2	8.34E-03	0.04375	Female

Table S15. KEGG pathways enriched in metabolism associated DEGs of male and female mouse offspring.

KEGG Pa	KEGG Pathway Term Desc	KEGG Pa	KEGG Pa	Term Can	Total Can	Term Ger	Total Ger	Rich Rati	P value	Q value	Remarks
330	Arginine and proline metaboli	Metabolisr	Amino aci	3	75	50	8491	0.06	9.64E-03	0.13041	Male
340	Histidine metabolism	Metabolisr	Amino aci	2	75	24	8491	0.08333	1.87E-02	0.19021	Male
524	Neomycin, kanamycin and ge	Metabolisr	Biosynthes	1	75	5	8491	0.2	4.34E-02	0.31466	Male
500	Starch and sucrose metaboli	Metabolisr	Carbohydr	3	75	33	8491	0.09091	2.99E-03	0.08658	Male
51	Fructose and mannose meta	Metabolisr	Carbohydr	3	75	35	8491	0.08571	3.54E-03	0.08974	Male
660	C5-Branched dibasic acid me	Metabolisr	Carbohydr	1	75	1	8491	1	8.83E-03	0.13041	Male
650	Butanoate metabolism	Metabolisr	Carbohydr	2	75	27	8491	0.07407	2.34E-02	0.20104	Male
590	Arachidonic acid metabolism	Metabolisr	Lipid meta	6	75	88	8491	0.06818	1.18E-04	0.01203	Male
72	Synthesis and degradation of	Metabolisr	Lipid meta	2	75	11	8491	0.18182	4.02E-03	0.0907	Male
565	Ether lipid metabolism	Metabolisr	Lipid meta	3	75	46	8491	0.06522	7.65E-03	0.13041	Male
120	Primary bile acid biosynthesis	Metabolisr	Lipid meta	2	75	16	8491	0.125	8.53E-03	0.13041	Male
600	Sphingolipid metabolism	Metabolisr	Lipid meta	3	75	48	8491	0.0625	8.61E-03	0.13041	Male
61	Fatty acid biosynthesis	Metabolisr	Lipid meta	2	75	19	8491	0.10526	1.19E-02	0.14883	Male
100	Steroid biosynthesis	Metabolisr	Lipid meta	2	75	20	8491	0.1	1.32E-02	0.14883	Male
900	Terpenoid backbone biosynth	Metabolisr	Metabolisr	2	75	23	8491	0.08696	1.73E-02	0.18458	Male
980	Metabolism of xenobiotics by	Metabolisr	Xenobiotic	3	75	67	8491	0.04478	2.12E-02	0.19539	Male
350	Tyrosine metabolism	Metabolisr	Amino aci	3	77	40	8491	0.075	5.57E-03	0.05537	Female
380	Tryptophan metabolism	Metabolisr	Amino aci	3	77	48	8491	0.0625	9.26E-03	0.08284	Female
280	Valine, leucine and isoleucine	Metabolisr	Amino aci	3	77	56	8491	0.05357	1.41E-02	0.11298	Female
360	Phenylalanine metabolism	Metabolisr	Amino aci	2	77	23	8491	0.08696	1.82E-02	0.13	Female
340	Histidine metabolism	Metabolisr	Amino aci	2	77	24	8491	0.08333	1.97E-02	0.13557	Female
250	Alanine, aspartate and glutar	Metabolisr	Amino aci	2	77	38	8491	0.05263	4.63E-02	0.28547	Female
232	Caffeine metabolism	Metabolisr	Biosynthes	2	77	6	8491	0.33333	1.19E-03	0.01638	Female
562	Inositol phosphate metabolisi	Metabolisr	Carbohydr	4	77	73	8491	0.05479	4.24E-03	0.04465	Female
1212	Fatty acid metabolism	Metabolisr	Global anc	4	77	61	8491	0.06557	2.21E-03	0.02821	Female
140	Steroid hormone biosynthesis	Metabolisr	Lipid meta	7	77	88	8491	0.07955	1.35E-05	4.83E-04	Female
591	Linoleic acid metabolism	Metabolisr	Lipid meta	5	77	49	8491	0.10204	7.51E-05	0.00224	Female
62	Fatty acid elongation	Metabolisr	Lipid meta	4	77	29	8491	0.13793	1.25E-04	0.00307	Female
590	Arachidonic acid metabolism	Metabolisr	Lipid meta	6	77	88	8491	0.06818	1.37E-04	0.00307	Female
1040	Biosynthesis of unsaturated f	Metabolisr	Lipid meta	3	77	32	8491	0.09375	2.94E-03	0.03513	Female
120	Primary bile acid biosynthesis	Metabolisr	Lipid meta	2	77	16	8491	0.125	8.97E-03	0.08284	Female
71	Fatty acid degradation	Metabolisr	Lipid meta	3	77	50	8491	0.06	1.04E-02	0.08825	Female
830	Retinol metabolism	Metabolisr	Metabolisr	9	77	91	8491	0.0989	1.11E-07	1.44E-05	Female
980	Metabolism of xenobiotics by	Metabolisr	Xenobiotic	5	77	67	8491	0.07463	3.35E-04	0.006	Female
982	Drug metabolism - cytochrom	Metabolisr	Xenobiotic	4	77	69	8491	0.05797	3.46E-03	0.03872	Female

Table S16. Molecular functions of apoptosis associated DEGs in male mouse and female offspring.

GO_F Ter	GO_F Term Desc	GO_F Ter	GO_F Ter	Term Can	Total Can	Term Ger	Total Gen	Rich Rati	P value	Q value	Remarks
GO:00162	antioxidant activity	molecular	antioxidan	4	83	27	20317	0.14815	4.23E-06	9.22E-04	Male
GO:00725	peroxynitrite reductase acti	molecular	antioxidan	1	83	1	20317	1	0.00409	0.04948	Male
GO:00082	zinc ion binding	molecular	binding	11	83	749	20317	0.01469	2.29E-04	0.01334	Male
GO:00356	Toll-like receptor 4 binding	molecular	binding	2	83	6	20317	0.33333	2.45E-04	0.01334	Male
GO:00507	RAGE receptor binding	molecular	binding	2	83	9	20317	0.22222	5.83E-04	0.02054	Male
GO:00505	arachidonic acid binding	molecular	binding	2	83	10	20317	0.2	7.26E-04	0.02054	Male
GO:00510	unfolded protein binding	molecular	binding	4	83	100	20317	0.04	7.54E-04	0.02054	Male
GO:00055	ATP binding	molecular	binding	15	83	1524	20317	0.00984	0.00119	0.02591	Male
GO:00518	Hsp90 protein binding	molecular	binding	3	83	51	20317	0.05882	0.00119	0.02591	Male
GO:00428	identical protein binding	molecular	binding	16	83	1718	20317	0.00931	0.00143	0.02831	Male
GO:00055	protein binding	molecular	binding	34	83	5245	20317	0.00648	0.00181	0.0303	Male
GO:00428	peptidoglycan binding	molecular	binding	2	83	18	20317	0.11111	0.00242	0.03514	Male
GO:00310	heat shock protein binding	molecular	binding	3	83	78	20317	0.03846	0.00401	0.04948	Male
GO:00046	protein kinase activity	molecular	catalytic a	8	83	549	20317	0.01457	0.0018	0.0303	Male
GO:00046	protein serine/threonine kin	molecular	catalytic a	7	83	433	20317	0.01617	0.00196	0.03057	Male
GO:00050	netrin receptor activity	molecular	molecular	2	83	6	20317	0.33333	2.45E-04	0.01334	Male
GO:00016	purinergic nucleotide recep	molecular	molecular	2	83	8	20317	0.25	4.54E-04	0.01981	Male
GO:00705	nicotinic acid receptor activ	molecular	molecular	1	83	1	20317	1	0.00409	0.04948	Male
GO:00055	protein binding	molecular	binding	46	86	5245	20317	0.00877	4.07E-08	5.01E-06	Female
GO:00037	double-stranded RNA bindi	molecular	binding	7	86	79	20317	0.08861	4.30E-08	5.01E-06	Female
GO:00468	metal ion binding	molecular	binding	35	86	3637	20317	0.00962	6.18E-07	4.80E-05	Female
GO:00507	RAGE receptor binding	molecular	binding	3	86	9	20317	0.33333	6.04E-06	3.52E-04	Female
GO:00017	2'-5'-oligoadenylate synthet	molecular	catalytic a	3	86	11	20317	0.27273	1.18E-05	4.89E-04	Female
GO:00051	tumor necrosis factor recep	molecular	binding	4	86	34	20317	0.11765	1.26E-05	4.89E-04	Female
GO:00428	identical protein binding	molecular	binding	20	86	1718	20317	0.01164	2.49E-05	8.30E-04	Female
GO:00036	left-handed Z-DNA binding	molecular	binding	2	86	3	20317	0.66667	5.30E-05	0.00154	Female
GO:00046	protein kinase activity	molecular	catalytic a	10	86	549	20317	0.01821	1.07E-04	0.00278	Female
GO:00356	Toll-like receptor 4 binding	molecular	binding	2	86	6	20317	0.33333	2.63E-04	0.00612	Female
GO:00036	DNA binding	molecular	binding	19	86	1924	20317	0.00988	3.63E-04	0.00712	Female
GO:00353	Toll-like receptor binding	molecular	binding	2	86	7	20317	0.28571	3.67E-04	0.00712	Female
GO:00448	protein-containing complex	molecular	binding	10	86	660	20317	0.01515	4.69E-04	0.00825	Female
GO:00046	protein serine/threonine kin	molecular	catalytic a	8	86	433	20317	0.01848	4.96E-04	0.00825	Female
GO:00051	cytokine activity	molecular	binding	6	86	241	20317	0.0249	5.58E-04	0.00864	Female
GO:00055	ATP binding	molecular	binding	16	86	1524	20317	0.0105	5.93E-04	0.00864	Female
